# Identification of Microbial Genetic Capacities and Potential Mechanisms Within the Rumen Microbiome Explaining Differences in Beef Cattle Feed Efficiency

**DOI:** 10.3389/fmicb.2020.01229

**Published:** 2020-06-05

**Authors:** Marc D. Auffret, Robert D. Stewart, Richard J. Dewhurst, Carol-Anne Duthie, Mick Watson, Rainer Roehe

**Affiliations:** ^1^Scotland’s Rural College (SRUC), Edinburgh, United Kingdom; ^2^Division of Genetics and Genomics, The Roslin Institute and R(D)SVS, University of Edinburgh, Edinburgh, United Kingdom; ^3^Edinburgh Genomics, The Roslin Institute and R(D)SVS, University of Edinburgh, Edinburgh, United Kingdom

**Keywords:** rumen microbiome, predicted microbial mechanisms, feed conversion efficiency, high concentrate diet, metagenomic sequencing

## Abstract

In this study, *Bos Taurus* cattle offered one high concentrate diet (92% concentrate-8% straw) during two independent trials allowed us to classify 72 animals comprising of two cattle breeds as “Low” or “High” feed efficiency groups. Digesta samples were taken from individual beef cattle at the abattoir. After metagenomic sequencing, the rumen microbiome composition and genes were determined. Applying a targeted approach based on current biological evidence, 27 genes associated with host-microbiome interaction activities were selected. Partial least square analysis enabled the identification of the most significant genes and genera of feed efficiency (VIP > 0.8) across years of the trial and breeds when comparing all potential genes or genera together. As a result, limited number of genes explained about 40% of the variability in both feed efficiency indicators. Combining information from rumen metagenome-assembled genomes and partial least square analysis results, microbial genera carrying these genes were determined and indicated that a limited number of important genera impacting on feed efficiency. In addition, potential mechanisms explaining significant difference between Low and High feed efficiency animals were analyzed considering, based on the literature, their gastrointestinal location of action. High feed efficiency animals were associated with microbial species including several *Eubacterium* having the genetic capacity to form biofilm or releasing metabolites like butyrate or propionate known to provide a greater contribution to cattle energy requirements compared to acetate. Populations associated with fucose sensing or hemolysin production, both mechanisms specifically described in the lower gut by activating the immune system to compete with pathogenic colonizers, were also identified to affect feed efficiency using rumen microbiome information. Microbial mechanisms associated with low feed efficiency animals involved potential pathogens within Proteobacteria and Spirochaetales, releasing less energetic substrates (e.g., acetate) or producing sialic acid to avoid the host immune system. Therefore, this study focusing on genes known to be involved in host-microbiome interaction improved the identification of rumen microbial genetic capacities and potential mechanisms significantly impacting on feed efficiency in beef cattle fed high concentrate diet.

## Introduction

The FAO predicted that by 2050, the human population will grow to over 9 billion people, and in the same time frame, global meat consumption is projected to increase by 73% (FAO, 2019). Meat production from ruminants offers several advantages including the fact that ruminants convert feedstuffs into high-quality nutrients from materials that do not compete with human-edible food.

The bovine rumen microbiome is essential for feed digestion and beneficial for the hosting ruminant animal. In addition to nutrient absorption by the host, digestion is recognized as an important source of variation in cattle growth efficiency (Herd et al., 2004; Roehe et al., 2016). Such variation is also diet dependent and the importance of diet in determining the composition of the ruminal microbiome is now well-recognized (Rooke et al., 2014; Henderson et al., 2015).

Although, intensive food production using more concentrate diet instead of forage improved feed efficiency in ruminant production, also reducing methane emissions, it generated a stress on the rumen microbiome and the animal (Auffret et al., 2017; Wetzels et al., 2017). For example, both studies confirmed the influence of concentrate over forage diets to generate rumen dysbiosis in beef and dairy cattle. This disturbance is explained by a breach of robustness in microbiome composition and functionality also associated with a “bloom” of zoonotic pathogens especially within Proteobacteria, as similarly, found in humans (Brown et al., 2012).

In nature, most microorganisms are known to occur predominantly in consortia or biofilms, even on mucosal surfaces, involving a broad range of mechanisms for adhesion on the mucosa by beneficial and detrimental microorganisms (Tuson and Weibel, 2013).

It is known that ruminal and lower gut epithelia are dramatically different in term of structure and type of cells with epithelial-attached microorganisms predominantly detected in the lower gut (Steele et al., 2016). Although limited research focused on epithelial-attached microorganisms in ruminant (Malmuthuge et al., 2015), it is predicted to indirectly impact animal performance (Steele et al., 2016). Furthermore, microbial genes detected in the rumen and associated with activities specific of the lower gut like fucose sensing were previously detected in rumen digesta samples (Roehe et al., 2016; Auffret et al., 2017). However, there is lack of information on which microbial mechanisms can impact on cattle feed efficiency. Therefore, identifying and studying such microbial genetic capacities detected in the rumen could be helpful to predict microbial mechanisms impacting animal feed efficiency.

Microbial biofilms have been studied in human (Macfarlane and Dillon, 2007) and ruminants (Bang et al., 2014) and are known to mediate protective functions and enables communication between biofilm forming microorganisms (Macfarlane and Dillon, 2007). Although in general, biofilm studies focused on detrimental effects of mucosa-associated bacterial pathogens such as *Escherichia*, *Shigella*, *Salmonella*, and *Treponema* spp. (Mao et al., 2015; Song et al., 2018), the presence of biofilms in the rumen has also been reported in healthy ruminants (Pitta et al., 2016).

Other possible microbial mechanisms involved the presence of pili or flagella for surface hooking, different types of secretion systems for adhesion and transfer of toxins or metabolites, production of enzymes needed for the biofilm matrix or associated with toxic effect on the host (e.g., hemolysin; Ribet and Cossart, 2015). Finally, the mucosa surface in the intestine is covered by mucin composed of several sugars including fucose but also sialic acids that can serve for the growth of commensal or pathogenic species (Pickard and Chervonsky, 2015). Alternatively, their synthesis by mucosa-attached microorganisms can assist in avoiding the immune system (Sicard et al., 2017).

To sustain beef cattle production the necessity to reduce production costs mostly associated with animal nutrition by improving feed utilization has been one of the main objectives over the last years (Ramsey et al., 2005). Feed efficiency is often assessed as either feed conversion ratio (FCR) or residual feed intake (RFI) with RFI considered more appropriate to generate a measure of biological efficiency independent of production (Koch et al., 1963; Gunsett, 1984). Negative values of both indicators are indicators of high feed efficiency.

The importance of the rumen microbiome as one of the factors impacting on animal feed efficiency is recognized. Previous reports mostly focused on the microbial community composition (16S rRNA gene data) showing that variation in animal feed efficiency was explained by particular taxa (Myer et al., 2015; Shabat et al., 2016) instead of using the entire microbiome that means the microbial community and their microbial genes. Li et al. (2009) and Roehe et al. (2016) showed that the host genome shapes the rumen microbiota and could be used to identify differences in feed conversion efficiency. From these studies, a substantial lack of mechanistic understanding remained due to the inherent limitation to get information on microbial activities when using 16S rRNA gene sequencing. To alleviate this limitation, Ross et al. (2013) suggested to use metagenomic sequence information to obtain improved predictive models for the use of the microbiome for animal breeding purposes. In addition, Roehe et al. (2016) demonstrated the advantages of using microbial genes as proxies for feed conversion efficiency.

The rumen is an anaerobic microbial ecosystem with the ability to convert carbohydrates to short-chain, volatile fatty acids (VFA), which are absorbed by the animal and used in energy metabolism and protein synthesis. Furthermore, dihydrogen (H_2_) is also formed as a result of fermentation, and it is central to microbial metabolic activities. However, it is used by methanogenic archaea to reduce CO_2_ to methane (CH4; Hungate, 1967) leading to the loss of feed gross energy, estimated at 2–12% (Johnson and Johnson, 1995). Therefore, it is expected that high efficient cattle should produce less methane.

To the best of our knowledge, only a limited number of studies like Lima et al. (2019) identified microbial gene biomarkers for FCR and RFI. These authors applied a non-targeted approach for the selection of genes from metagenomics data. In the present study, a specific approach was applied selecting genes encoding for activities related to host–microbiome crosstalk and bacterial mobility and associating those with FCR and RFI and determine the microbial taxa carrying these genes based on metagenome-assembled genomes (MAGs) obtained by metagenomic sequencing (Stewart et al., 2018).

We hypothesized that differences observed in feed efficiency in apparently healthy cattle offered a high concentrate-based diet can be partly explained by differences in the rumen microbiome, particularly in microbial genera carrying genes related to adhesion and host-microbiome interaction. Rumen samples enriched in microbial genes known to be related to pathogenicity activities are detected in less feed efficient cattle. Genes carried by commensal bacteria associated with beneficial mechanisms occurring in the rumen but also those genes which mechanisms is explained for the lower gut (e.g., fucose sensing) are detected in high feed efficient cattle.

Therefore, the overall aim of our work was to identify important microbial genera and potential mechanisms in animal fed concentrate diet, having the capacity to explain differences in animal feed efficiency by calculating feed conversion ratio and residual feed intake. Importance of such mechanisms impacting on feed efficiency was quantified using statistical model. This could be an important step toward discrimination between beneficial and detrimental microorganisms both carrying genes associated with host interaction and to develop strategies targeting microorganisms with the aim of increasing animal feed efficiency using breeding or dietary intervention.

## Materials and Methods

### Ethics Statement

The animal experiment was conducted at the Beef and Sheep Research Centre of Scotland’s Rural College (SRUC, Edinburgh, United Kingdom). The experiment was approved by the Animal Experiment Committee of SRUC and was conducted in accordance with the requirements of the UK Animals (Scientific Procedures) Act 1986.

### Animals, Experimental Design, and Animal Grouping

In our previous studies (Duthie et al., 2016, 2017), data on feed efficiency were obtained from a 2 × 2 factorial design experiment of breed types and diets using 72 steers each in two trial from purebred Luing and crossbred Charolais (CHx) steers. These animal trials were completed in 2012 and 2013 and more details are described in Supplementary Table S1 and Duthie et al. (2016, 2017). Prior to start of the experiment, all animals received the same diet type (Forage-based diet) and thereafter allocated to two different diets fed *ad libitum* containing (g/kg DM) ∼80 straw to 920 concentrate or 520 forage and 480 concentrate considered as high concentrate-based diet and a mixed diet, respectively. After a 5-week adaptation period to the diets, a 56-day test period for feed efficiency was carried out. Due to EU legislation, the application of antibiotics is prohibited for enhancing growth. In exceptional cases, animals were treated with antibiotics and then excluded from the trial. Information on the diets used in these animal trials was described in Duthie et al. (2016, 2017). Animals were fed *ad libitum*, the same allocated diet during adaptation period to the feed until they were slaughtered in the abattoir. There was no fastening period prior to sending the animals to slaughter. Gas emissions including methane were measured individually for 48 h in respiration chambers following the same procedure described in Rooke et al. (2014). Sample of ruminal digesta used to determine the microbiome were taken at slaughter between 4 and 14 weeks after the end of the feed efficiency monitoring period. This time gap between the end of the feed efficiency period and the collect of the rumen samples at the abattoir was due to monitoring of methane emissions in six available respiration chambers in which all cattle were recorded individually.

The methodology applied to collect the rumen digesta samples at the abattoir followed the same procedures previously described (Wallace et al., 2015; Roehe et al., 2016). Briefly, two rumen digesta (a mixed of solid and fluid) samples (~50 ml) were taken immediately after the rumen was opened to be drained prior to be stored at −80°C. The slaughter process results in well-mixed samples of rumen contents.

Within this study, only animals (*n* = 72) receiving high concentrate-based diet within the two independent beef cattle trials of crossbred Charolais and purebred Luing were used.

**FIGURE 1:**
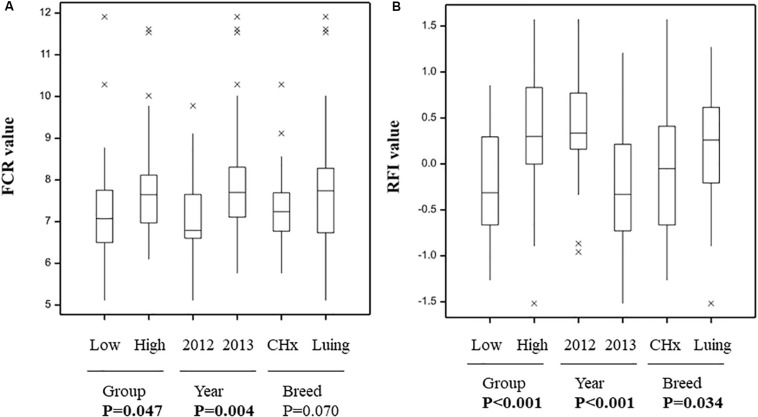
Variation in feed conversion ratio **(A)** and residual feed intake **(B)** between animals grouped based on feed efficiency indicators year and breed. *P*-value as indicator of significant difference (in bold when *P* < 0.05) between Low and High efficient animals.

The two feed efficiency indicators were calculated as follow: FCR is the ratio of feed intake to weight gain and provides an indication of the animal’s ability to convert feed to body weight. RFI is an estimation of the difference between actual feed intake and a predicted feed intake based on body weight and production following Savietto et al. (2014) calculation. Within 72 animals from 2 independent animal trials, half animals were classified as “Low” or “High” feed efficiency groups (Supplementary Table S1). The grouping between Low and High animals followed a balance designed including year of the animal trial (2012 and 2013) and breed (CHx and Luing). Significant differences in FCR and RFI between Low and High animal groups were subsequently confirmed (Figure 1). Immediately after the steers (within 2 h) left the respiration chambers, samples of ruminal fluid were obtained (one per animal) by inserting a tube (16 × 2700 mm Equivet Stomach Tube; Jørgen Kruuse A/S) nasally and aspirating manually. Approximately 50 ml of the fluid were strained through two layers of muslin and then deproteinised by adding 0.2 ml of metaphosphoric acid (215 g/l) and 0.1 ml of internal standard (10 ml 2-ethyl n-butyric acid/l) to determine volatile fatty acid (VFA) concentrations by HPLC analysis (1 ml) as described in Rooke et al. (2014).

### Metagenomics Annotation and Analysis

To get information on the rumen microbial communities and their genes, all rumen samples were collected at the abattoir as previous results confirmed high microbiome similarity between rumen fluid samples collected using stomach tube and rumen digesta collected at the abattoir (Wallace et al., 2014; Snelling et al., 2019). DNA was extracted from the rumen digesta samples following the protocol from Yu and Morrison (2004) and was based on repeated bead beating with column filtration. The procedure is fully described in Rooke et al. (2014).

Illumina TruSeq libraries were prepared from genomic DNA and sequenced on an Illumina HiSeq 4000 instrument by Edinburgh Genomics (Edinburgh, United Kingdom). Paired-end reads (2 × 100 bp) were generated, resulting in between 8 and 15 GB per sample (between 40 and 73 million paired reads) with on average 73% passing quality check and being subsequently annotated. Bioinformatics analysis followed the same procedure as previously described in Roehe et al. (2016) and Wallace et al. (2015). In order to measure the abundance of known microbial genes in the rumen samples, reads from whole metagenome sequencing were aligned to the Kyoto Encyclopedia of Genes and Genomes (KEGG)^1^ database. The KEGG Orthologue groups (KO) of all hits that were equal to the best hit were examined. In the case we were unable to resolve the read to a single KO, the read was ignored; otherwise, the read was assigned to the unique KO. Statistical analysis of the metagenomics samples was based on the complete sample profiles as expressed by the pattern of metagenomic reads classified within KEGG orthologue groups with >90% similarity and belonging to a single KEGG orthologue (KO) groups. The alignment of the reads generated by whole metagenomic sequencing to the KEGG genes database resulted in identification of 4,427 microbial genes for each animal. Microbial genes were expressed in relative abundance (percentage) within animal and only those with a relative abundance greater than 0.001% (*n* = 1,630) were carried forward for downstream analysis.

For phylogenetic annotation, the genomic reads were aligned to a custom database using Kraken combining several databases including genomes from the Hungate 1,000 collection and metagenome-assembled genomes (MAGs) from beef rumen samples (Wood and Salzberg, 2014; Stewart et al., 2018). All taxonomic labels identified are subsequently described as the genus having the highest similarity with the identified genome or MAG and applying the same cutoff used in previous MAG study (Stewart et al., 2018) (estimated completeness ≥ 80% and estimated contamination ≤ 10%). As for microbial genes, microbial genera identified (*n* = 1,058) were normalized between animals expressing them as relative abundances only those with a relative abundance greater than 0.001% (*n* = 1,630) were carried forward for downstream analysis. The absence of “0” abundancies within the dataset was confirmed.

Following this step, 27 genes (within 1,630 genes), 42 phyla and 1,058 genera were selected for the statistical analysis. These microbial genes were selected based on their functions associated with different microbiome-host mucosa interaction mechanisms including flagella, pilus, secretion system, biofilm formation and fucose or sialic acid metabolism in order to answer to our hypotheses. The 27 genes were selected based on biological evidence that bacteria carrying such genes have the capacity to interact with the host. Based on our hypothesis, such genetic capacities could potentially affect animal performance in term of feed efficiency.

Information on the gene content within metagenome-assembled genomes (Stewart et al., 2018) was used to validate the microbial genera identified as highly important to explain variation in animal feed efficiency. MAGs with the highest % of similarity and query coverage using the Genome Taxonomy Database (GTDB) were selected as best hit and the most probable bacterial taxa carrying each particular gene studied.

Metagenomics raw sequencing data combined with metadata of the animal experiments can be downloaded from the European Nucleotide Archive under accession PRJEB10338 and PRJEB31266.

### Statistical Analysis

Methodologies to analyze metagenomics data by General Linear Model (GLM; including year of the trial and breed type as fixed effects), Principal Coordinate Analysis (PCoA) and Canonical Variate Analysis (CVA) using Gen-Stat 16th edition (VSN International Ltd., United Kingdom), and Partial Least Square (PLS) analysis using SAS (Version 9.1 for Windows, SAS Institute Inc., Cary, NC, United States) were similar to those described in Auffret et al. (2017). Results were considered as significantly different when the *P*-value was *P* < 0.05. In order to identify the influence of microbial variables (genes or genera) and potential mechanisms that explained most of the variation in FCR and RFI, different Partial Least Square (PLS) models per gene were performed. Each model was built considering RFI or FCR as dependent variables, year and breed as fixed effects and each microbial gene as explanatory variables (Script in Supplementary Data). The most influential microbial genes or genera from each model that were important in explaining RFI or FCR were selected based on the variable importance for projection (VIP) criterion (Wold, 1995) whereby microbial genes with a VIP <0.8 contribute little to the prediction.

PLS analysis was first applied to identify the genes within the 27 preselected genes mostly explaining variation in FCR or RFI. This methodology was successfully applied to identified microbial biomarkers explaining variation in several beef cattle traits as shown in Auffret et al. (2017, 2018) and Lima et al. (2019). Secondly, a similar analysis was applied to determine the microbial genera mostly explaining variation in FCR or RFI. In parallel, using data from Stewart et al. (2018) the MAGs carrying the genes identified by PLS explaining variation in FCR or RFI were identified and such information was combined with the genera results to determine the microbial taxa carrying one particular gene identified as important to explain variation in RFI or FCR. Importance of individual VFA and acetate-to-propionate ratio was investigated using PLS analysis (breed and year as fixed effects) in addition to the 27 selected microbial genes. The acetate-to-propionate ratio was calculated and considered as a proxy for rumen pH, accepting that whilst the relationship between the two is generally strong, it is not exactly linear (Russell, 1998).

A Venn diagram was generated using Venny software (Oliveros, 2007) to compare the similarity in term of microbial genes or genera explaining most of the variability in FCR or RFI.

In addition, Linear Discriminant Analysis (LDA) using the LDA function in R (version 3.5.1.) was applied on 13 microbial genes and 128 microbial genera, all identified by PLS as important to explain the variation observed in animals with significantly different RFI and FCR values.

A prediction accuracy value (%) was calculated as indicator of accurate identification of Low or High feed efficiency animals based on the selected genes or genera.

## Results

### Differences in Microbial Activities and Host Feed Efficiency Between Groups of Differently Feed Efficiency Animals

In this study, “High” animals have a low FCR and RFI values meaning they are efficient to convert feed into live weight gain, whilst “Low” animals performing in the opposite way.

All animals offered concentrate diet were selected and showed significant differences between groups of animals based on FCR or RFI (Figure 1), also confirming the grouping selection based on feed efficiency indicators and balanced for year of the trial and breed (Supplementary Table S1). Furthermore, significant differences between groups of animals based on years of the trial and breeds were found (Figure 1). Only the differences between breeds for FCR showed a tendency (*P* = 0.07).

Microbial activities associated with gas production including methane emissions (Supplementary Figure S1) or VFA (Supplementary Figure S2) were not significantly different between Low and High animals and the acetate-to-propionate ratio used as a proxy for rumen pH was not significantly different neither between the two groups of animals (Supplementary Figure S2C).

### Variation in the Rumen Microbiome Composition Between Low and High Feed Efficiency Animals

**FIGURE 2:**
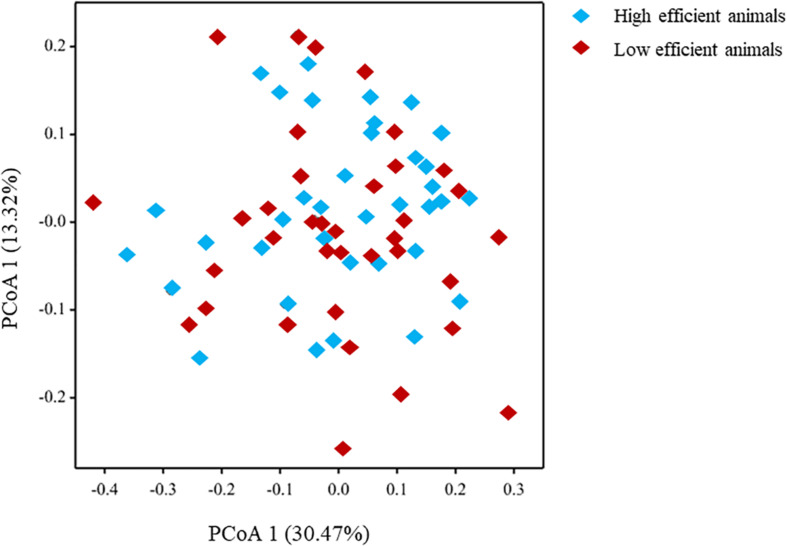
Principal Coordinate Analysis (PCoA) of the microbiome community using microbial genera.

The microbial community composition studied at the genus level showed a limited difference using Principal Coordinate Analysis (PCoA) between animals identified with Low- compared to High feed efficiency (Figure 2). This result was confirmed by Canonical Variate Analysis (CVA) as indicated by the overlapping of the 95% confidence circles between the two animal groups (Supplementary Figure S3).

Within 1,058 genera with a relative abundance above 0.001%, 379 genera were found significantly different (*P*< 0.05) by GLM based on the samples grouping (Supplementary Table S2). Genera within Proteobacteria phylum represent 35% of the 379 genera (Supplementary Figure S4) followed by genera within Firmicutes (16%) and Actinobacteria (17%). Within the 132 Proteobacteria genera, 32 were significantly higher in the Low feed efficiency group whilst 100 were dominant (*P* < 0.05) in the other group (Supplementary Table S2). Within Firmicutes and Bacteroidetes, *Prevotella* (Bacteroidetes) was the most dominant species significantly higher in the Low group like the Firmicutes genera *Eubacterium*, *Sharpea*, *Clostridium*, *Lactobacillus*, and *Paenibacillus*. *Bifidobacterium* and *Ilumatobacter* (Actinobacteria) were also significantly higher in the Low group. In the High feed efficiency group, genera associated with metabolic activities such as *Succiniclasticum* and *Succinivibrio* (succinate), *Acidaminococcus* (amino acids), *Sarcina* and *Fibrobacter* (cellulose) were all significantly more abundant.

Genera associated with *Staphylococcus* (Firmicutes), the protist *Eimeria* (Alveolata), *Sphaerochaeta* and *Treponema* (Spirochaetes), *Vibrio* and *Lawsonia* (Proteobacteria) known to be potential pathogens were all significantly more abundant in the High group.

### Selected Rumen Microbiome Genes and Potential Mechanisms Explaining Variation in FCR and RFI

Genes selected for this study and associated with biofilm formation (K01335 and K01840), secretion system (K02005) and hemolysin synthesis were found significantly (*P* < 0.05) higher in the Low group by GLM analysis (Table 1). On the other hand, genes associated with sialic acid production (K01654), secretion systems (K01993 and K02454), pilus (K02283, K02652, K02653, K02662, and K02666), and flagellum (K02412), were significantly more abundant in High animals (Table 1).

**TABLE 1:** General linear Model (GLM) analysis on the selected microbial genes.

**KEGG ID gene**	**General function**	**Protein**	**GIT^a^ location of possible impact**	**Mean abundance LOW**	**Mean abundance HIGH**	***F*-value**	***P*-value**
K01206	Fucose sensing	Alpha-L-fucosidase	Intestine	0.012	0.010	1.187	0.325
**K01654**	Mucus interaction	N-acetylneuraminate synthase	Rumen and intestine	0.0020	0.0021	3.425	**0.013**
K01818	Fucose sensing	L-fucose isomerase	Intestine	0.014	0.015	0.92	0.458
**K01835**	Biofilm formation	Phosphoglucomutase	Rumen and intestine	0.005	0.001	3.429	**0.013**
**K01840**	Biofilm formation	Phosphomannomutase	Rumen and intestine	0.277	0.250	4.989	**0.001**
**K01993**	Secretion system	HlyD family secretion protein	Rumen and intestine	0.0137	0.0145	2.806	**0.032**
K02005	Secretion system	HlyD family secretion protein	Rumen and intestine	0.118	0.099	2.101	0.09
**K02283**	Pilus	Pilus assembly protein CpaF	Rumen and intestine	0.0068	0.0071	4.431	**0.003**
K02377	Fucose sensing	GDP-L-fucose synthase	Intestine	0.034	0.034	1.181	0.327
K02390	Flagella	Flagellar hook protein FlgE	Rumen and intestine	0.001	0.001	1.91	0.119
K02392	Flagella	Flagellar basal-body rod protein FlgG	Rumen and intestine	0.002	0.003	1.533	0.203
K02396	Flagella	Flagellar hook-associated protein 1 FlgK	Rumen and intestine	0.001	0.001	1.436	0.232
K02400	Flagella	Flagellar biosynthesis protein FlhA	Rumen and intestine	0.006	0.008	0.886	0.477
K02406	Flagella	Flagellin	Rumen and intestine	0.011	0.013	1.356	0.259
K02407	Flagella	Flagellar hook-associated protein 2	Rumen and intestine	0.005	0.007	0.468	0.759
K02410	Flagella	Flagellar motor switch protein FliG	Rumen and intestine	0.001	0.002	0.868	0.488
**K02412**	Flagella	Flagellum-specific ATP synthase	Rumen and intestine	0.001	0.002	4.559	**0.003**
K02429	Fucose sensing	MFS transporter, FHS family, L-fucose permease	Intestine	0.031	0.025	0.864	0.49
**K02454**	Secretion system	General secretion pathway protein E	Rumen and intestine	0.005	0.006	11.788	**0.001**
**K02652**	Pilus	Type IV pilus assembly protein PilB	Rumen and intestine	0.010	0.015	8.995	**0.001**
**K02653**	Pilus	Type IV pilus assembly protein PilC	Rumen and intestine	0.004	0.006	10.751	**0.001**
K02662	Pilus	Type IV pilus assembly protein PilM	Rumen and intestine	0.002	0.002	2.048	0.098
K02666	Pilus	Type IV pilus assembly protein PilQ	Rumen and intestine	0.003	0.006	2.113	0.089
K03205	Secretion system	Type IV secretion system protein VirD4	Rumen and intestine	0.007	0.011	1.458	0.225
**K06442**	Virulence	Putative hemolysin	Intestine	0.0033	0.0031	4.155	**0.005**
K11068	Virulence	Hemolysin III	Intestine	0.004	0.003	2.069	0.095
K11907	Secretion system	Type VI secretion system protein VasG	Rumen and intestine	0.011	0.013	1.564	0.194

*^*a*^GIT: Gastrointestinal tract with two locations identified as rumen or intestine. Year of animal trial and Breed were included as fixed effect in the GLM analysis. Bold: Genes with a relative abundance significantly different (P < 0.05) between Low and High feed efficiency (both FCR and RFI) animal groups.*

**FIGURE 3:**
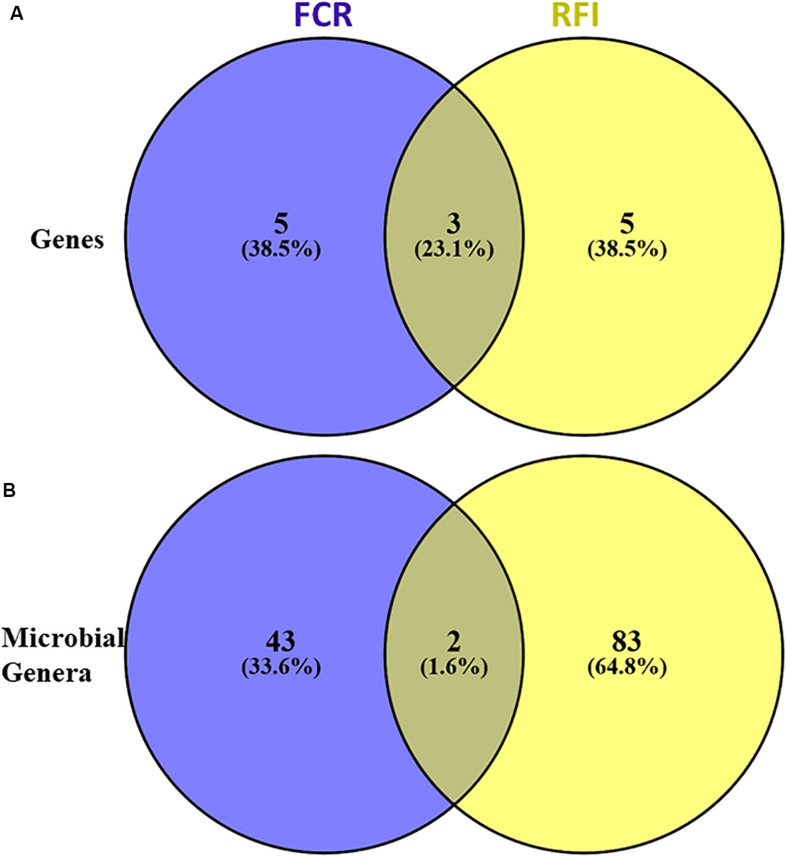
Venn diagram representing the microbial genes **(A)** and genera **(B)** associated with feed conversion ratio (FCR) and residual feed intake (RFI).

Eight microbial genes explained 39 and 40% of the variability in FCR and RFI, respectively, as determined by Partial Least Square (PLS) analysis (Table 2). Genes associated with biofilm formation (K01840), Type I secretion system (K02005), hemolysin synthesis (K11068) and fucose sensing (K02429) showed a negative correlation with FCR whilst two genes encoding for general and Type IV of secretion systems (K02454 and K03205) and two genes encoding for Type IV pilus (K02652 and K02653) were positively correlated with FCR. Genes K02454, K02652, and K02653 were also found correlated with RFI (Figure 3A). Other genes positively correlated with RFI encoded for sialic acid synthesis (K01654) and flagellum (K02410) whilst those negatively correlated with RFI were associated with pilus assembly protein (K02283), flagellar hooking protein (K02396), and hemolysin synthesis (K06442).

**TABLE 2:** Partial Least Square results for the microbial genes explaining the variability in feed conversion ratio and residual feed intake.

**KEGG ID gene**	**General function**	**Protein**	**VIP**	**Coefficient**
**Feed conversion ratio (FCR)**				
K01840	Biofilm formation	Phosphomannomutase	0.89	−0.02
K02005	Secretion system	HlyD family secretion protein	0.93	−0.05
K02429	Fucose sensing	MFS transporter, FHS family, L-fucose permease	0.92	−0.14
K02454	Secretion system	General secretion pathway protein E	0.87	0.04
K02652	Pilus	Type IV pilus assembly protein PilB	0.88	0.02
K02653	Pilus	Type IV pilus assembly protein PilC	0.83	0.00
K03205	Secretion system	Type IV secretion system protein VirD4	1.59	0.29
K11068	Virulence	Hemolysin III	0.92	−0.16
**Residual feed intake (RFI)**				
K01654	Sialic acid synthesis	N-acetylneuraminate synthase	0.77	0.09
K02283	Pilus	pilus assembly protein CpaF	0.83	−0.02
K02396	Flagella	Flagellar hook-associated protein 1 FlgK	0.74	−0.07
K02410	Flagella	Flagellar motor switch protein FliG	1.17	0.21
K02454	Secretion system	General secretion pathway protein E	0.91	0.00
K02652	Pilus	Type IV pilus assembly protein PilB	1.03	−0.14
K02653	Pilus	Type IV pilus assembly protein PilC	0.83	0.00
K06442	Virulence	Putative hemolysin	0.84	−0.04

*VIP, Variable Importance for Projection obtained by PLS analysis.*

Inclusion of VFA data and the acetate-to-propionate ratio as proxy for the rumen pH into the PLS analysis in addition to the 27 selected genes did not improve the percentage of variation explained by the model for FCR and RFI, as indicated by VIP values of these additional variables being in general below 0.8 except for branched chain fatty acids showing a negative effect on RFI (Supplementary Table S3).

### Identification of Potential Microbial Species Carrying Studied Genes and Explaining Variation in FCR and RFI

Using a PLS approach, a list of genera found highly correlated (VIP > 0.8) with the selected genes was identified (Supplementary Table S4) and this list was refined using gene content information from the MAGs previously generated in Stewart et al. (2018). In parallel, 45 and 85 genera were also found significantly correlated by PLS with FCR or RFI, respectively (Figure 3B), also explaining 60 and 52% of the variability observed in FCR and RFI, respectively (Supplementary Table S5).

Only two genera associated with *Gordonibacter* (Actinobacteria) and *Sarcina* (Firmicutes) were found both correlated with FCR and RFI.

**FIGURE 4:**
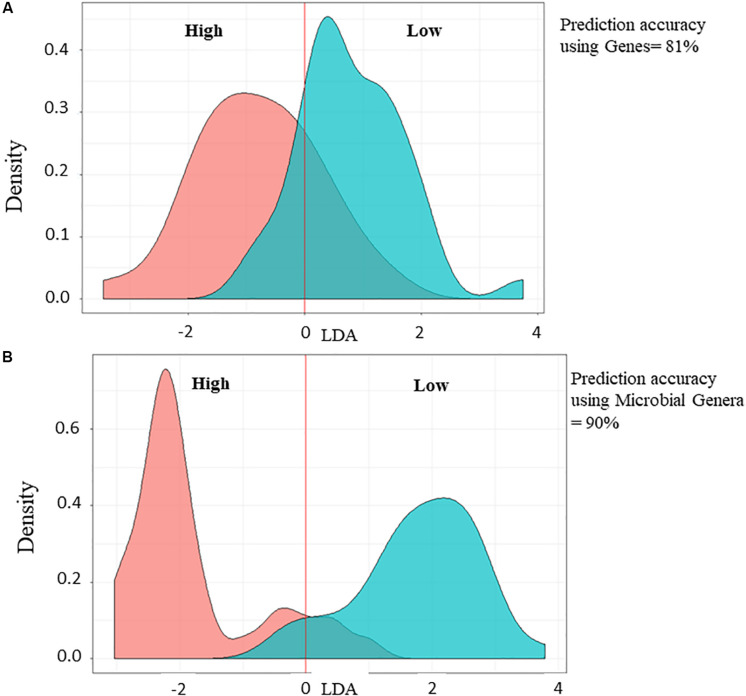
Prediction accuracy analysis using Linear Discriminant Analysis for the validation of the selected microbial genes **(A)** and genera **(B)**. Low and High feed efficiency animals are represented in green curve and red curve, respectively. Percentage of prediction accuracy is indicated.

In parallel to PLS analysis for the identification of biomarkers associated with FCR or RFI, LDA was applied to determine the prediction accuracy of Low compared to High feed efficiency animals and showed 81 or 90% of prediction accuracy using 13 genes (Figure 4A) or 148 microbial genera (Figure 4B), respectively.

On average, 22 (±8) genera were significantly correlated with each gene and explained on average 52 ± 8% of the variability observed per gene. Over the 37 genera identified as highly correlated with at least one of the selected genes showing high correlation with FCR and/or RFI, 19 genera belonged to Proteobacteria and 8 Proteobacteria genera correlated with one of the genes showed a beneficial effect on feed efficiency (negative coefficient value). Genera within Actinobacteria (4/19) and Firmicutes (6/19) were also identified as important populations carrying genes related to adhesion activities.

The genera related to *Eubacterium* (Firmicutes) were found highly correlated with four genes encoding for the production of pilus (K02283 and K02652), flagella (K02396) and a putative hemolysin (K06442) and always negatively correlated with RFI (Table 4). Although *Eubacterium* was composed of 10 different species or variants based on GTDB database (Supplementary Table S6), species Q, H, and A were the most abundant constituting *Eubacterium*. Such diversity of species identified as important to explain variation in host feed efficiency was not observed in the other genera in our results (Supplementary Table S6).

Other genera identified as *Succiniclasticum* (Firmicutes, K01840 and K2005), *Lactobacillus* (Firmicutes, K02429), and *Bifidobacterium* (Actinobacteria, K11068) were all found negatively correlated with FCR or RFI. On the other hand, some genera were found highly correlated by PLS with two genes and also positively correlated with FCR or RFI (Tables 3, 4). For example, *Desulfococcus* and *Variovorax* within Proteobacteria and *Sphaerochaeta* (Spirochaetes) with genes involved in secretion system (K02454) and type IV pilus (K02653); the Spirochaetes *Treponema* was found positively correlated with FCR and genes involved in the formation of Type IV pilus (K02652) or secretion system (K03205). Finally, several genera within Proteobacteria were all found positively correlated with RFI and also highly correlated with genes encoding for sialic acid synthesis (K01654 with *Lawsonia* and *Succinivibrio*), pilus formation (K02283 with *Pannonibacter*) and flagella formation (K02410 with *Providencia*).

**TABLE 3:** Identification of microbial genera (including MAGs) and genes significantly correlated with residual feed intake.

**Domain**	**Genus**	**Mean low**	**Mean high**	**VIP^a^**	**Coef.^a^**	**Gene^b^**
**Residual feed intake**						
Fungi	*Fibroporia*	0.001	0.001**	1.167	0.041	K01654
Bacteria	*Idiomarina*	0.001	0.002**	1.044	0.038	K01654
Bacteria	*Lawsonia*	0.002	0.003*	0.856	0.031	K01654
Bacteria	*Paraglaciecola*	0.001	0.001	1.072	0.039	K01654
Bacteria	*Eubacterium*	1.524**	1.437	0.854	–0.027	K02283
Bacteria	*Defluviimonas*	0.007	0.007	0.917	–0.007	K02283
Bacteria	*Agrobacterium*	0.039**	0.038	0.951	0	K02283
Bacteria	*Pannonibacter*	0.008	0.009	0.949	0	K02283
Bacteria	*Eubacterium*	1.524**	1.437	0.854	–0.027	K02396
Bacteria	*Azorhizobium*	0.013**	0.012	0.885	–0.002	K02396
Bacteria	*Cryptobacterium*	0.007	0.005	0.892	–0.03	K02396
Fungi	*Glarea*	0.003	0.004**	0.830	–0.013	K02396
Bacteria	*Pantoea*	0.042	0.045**	0.911	–0.006	K02396
Bacteria	*Porphyrobacter*	0.007	0.007	0.902	–0.017	K02396
Bacteria	*Rhodomicrobium*	0.005	0.005	0.847	–0.009	K02396
Bacteria	*Providencia*	0.006	0.008	1.136	0.037	K02410
Bacteria	*Robiginitalea*	0.009	0.01	1.121	0.036	K02410
Bacteria	*Acidaminococcus*	0.971	1.051**	0.928	0.001	K02454
Bacteria	*Arthrobacter*	0.008	0.011	1.06	0.01	K02454
Bacteria	*Desulfococcus*	0.020	0.022*	1.205	0.024	K02454
Bacteria	*Rhizobium*	0.091	0.091*	0.945	0.001	K02454
Bacteria	*Variovorax*	0.027	0.028**	0.955	0.001	K02454
Bacteria	*Eubacterium*	1.524**	1.437	0.854	–0.027	K02652
Bacteria	*Methylomicrobium*	0.010	0.011**	0.81	–0.01	K02652
Bacteria	*Thermosynechococcus*	0.003**	0.002	1.094	–0.04	K02652
Bacteria	*Acidaminococcus*	0.971	1.051**	0.928	0.001	K02653
Bacteria	*Brevundimonas*	0.020	0.021**	1.044	0.01	K02653
Bacteria	*Eubacterium*	1.524**	1.437	0.854	–0.027	K06442

VIP, Variable Importance for Projection; Coef., Coefficient associated with the VIP value obtained by PLS analysis. ^*a*^PLS results between microbial genera and FCR or RFI. ^*b*^Genes identified as highly correlated with the microbial genera by PLS and also identified in the genome of the corresponding MAG. Bold: Significant GLM results for microbial genera with year and breed as fixed effect; *P < 0.05; **P < 0.001.

**TABLE 4:** Identification of microbial genera (including MAGs) and genes significantly correlated with feed conversion ratio.

**Domain**	**Genus**	**Mean low**	**Mean high**	**VIP^a^**	**Coef.^a^**	**Gene^b^**
**Feed conversion ratio**						
Bacteria	*Succiniclasticum*	5.054	5.433*	0.820	-0.021	K01840
Bacteria	*Succiniclasticum*	5.054	5.433*	0.820	-0.021	K02005
Bacteria	*Lactobacillus*	0.417*	0.405	0.654	-0.002	K02429
Bacteria	*Acidaminococcus*	0.971	1.051**	1.033	0.046	K02454
Bacteria	*Magnetospira*	0.004	0.004*	1.127	0.054	K02454
Bacteria	*Salinicoccus*	0.003	0.004*	1.177	0.055	K02454
Bacteria	*Scardovia*	0.002	0.003	1.046	0.053	K02454
Bacteria	*Sphaerochaeta*	0.021	0.024**	1.11	0.037	K02454
Bacteria	*Acetobacterium*	0.006	0.006	1.094	0.032	K02454
Bacteria	*Treponema*	0.635	0.790**	1.077	0.061	K02652
Bacteria	*Acidaminococcus*	0.971	1.051**	1.033	0.046	K02653
Bacteria	*Sphaerochaeta*	0.0210	0.024**	1.110	0.037	K02653
Bacteria	*Treponema*	0.635	0.790**	1.077	0.061	K03205
Bacteria	*Elusimicrobium*	0.002	0.004*	1.148	0.067	K03205
Protozoa	*Trichomonas*	0.025	0.041	0.895	0.055	K03205
Bacteria	*Bifidobacterium*	0.942*	0.372	0.868	-0.003	K11068
Bacteria	*Methyloversatilis*	0.004	0.004	1.009	-0.06	K11068
Bacteria	*Phenylobacterium*	0.007	0.007	0.822	-0.051	K11068

VIP, Variable Importance for Projection. Coef., Coefficient associated with the VIP value obtained by PLS analysis. ^*a*^PLS results between microbial genera and FCR or RFI. ^*b*^Genes identified as highly correlated with the microbial genera by PLS and also identified in the genome of the corresponding MAG. Bold: Significant GLM results for microbial genera with year and breed as fixed effect; *P < 0.05; **P < 0.001.

Some other genera known to be involved in metabolite synthesis like the amino acid degrader *Acidaminococcus* (Firmicutes) was found positively correlated with FCR and RFI and also highly correlated with genes for the formation of secretion system (K02454) and Type IV pilus (K02653). Bacterial genera like *Azorhizobium* or *Agrobacterium* (both Proteobacteria) are generally interacting with plant tissue and in this study found highly correlated with flagella (K02396) or pilus (K02283) formation, respectively.

## Discussion

This is one of the first reports that identified and demonstrates the importance of particular rumen microbial genetic capacities significantly affecting animal feed efficiency using a large dataset from two independent experiments.

### Contribution of the Rumen Microbial Communities in Low and High Feed Efficient Beef Cattle

It is already well-known that inter-animal variation in feed efficiency exists among cattle of different breeds and receiving the same diet (Crowley et al., 2010; Cantalapiedra-Hijar et al., 2018). As a result, production cost can increase and was calculated in the current study (ca. on average 71 ± 32 Ł/animal between efficient and less efficient animals). Furthermore, grouping animals between low and high feed efficiency can help identifying possible microbial mechanisms explaining such variation (Roehe et al., 2016).

Our hypothesis was that differences observed in feed efficiency in cattle offered a high concentrate-based diet can be linked to differences in the rumen microbiome. Such animal-to-animal variation in feed efficiency was not significantly explained by differences in the entire rumen microbiota structure in agreement with previous work (reviewed in Cantalapiedra-Hijar et al., 2018). However, significant differences in feed efficiency between animal groups were mainly explained by a limited number of microbial genes and genera.

Some of the identified genera (e.g., *Succiniclasticum, Eubacterium*) were among the most dominant within the communities, partly contrasting with previous results indicating a lack of correlation between abundant taxa and feed efficiency (Hernandez-Sanabria et al., 2010, 2012). A possible explanation was a better characterization of the rumen microbiome following the recent advances in bioinformatics and culturomics (Seshadri et al., 2018; Stewart et al., 2018) and the release of thousands of new MAGs and complete rumen genomes used in this study. Furthermore, this study took advantages of such knowledge to identify the impact of microbial genes carried with certainty in the genome of rumen bacterial genera on host feed efficiency.

### Importance of Microbial Biomarkers Explaining Differences in Feed Efficiency in Beef Cattle

Contrasting with previous reports mostly using microbial community composition data (16S rRNA gene sequencing), we used microbial gene information from metagenomics sequencing data to study the link between microbial genetic capacities and animal feed efficiency. Using a non-targeted approach, Lima et al. (2019) identified a limited number of microbial genes explaining between 63 and 65% of the variability in FCR and RFI. In this study, a limited number of microbial genes selected based on previous biological evidence (Pickard and Chervonsky, 2015; Ribet and Cossart, 2015; Pitta et al., 2016; Auffret et al., 2017) allowed to explain about 40% of variability in FCR and RFI.

It is known that high concentrate diets can induce gastrointestinal dysbiosis including acidosis (Nagaraja et al., 1998) and an increase in pathogenic bacteria carrying genes associated with host mucosa interaction and pathogenicity mechanisms (Auffret et al., 2017; Sicard et al., 2017) in some animals. Although most of the work on rumen dysbiosis is related to cattle health, we hypothesized that such microbial genetic capacities, enriched in the rumen microbiome of animals more susceptible to high concentrate diet, are one of the potential reasons leading to an overall reduction in animal performance (Ramsey et al., 2005; Qin et al., 2010; Elolimy et al., 2018).

The combination of different bioinformatic and modeling methodologies used in this study helped to identify potential microbial genetic capacities and mechanisms significantly affecting beef cattle feed efficiency. In addition, the use of both FCR and RFI was advantageous to identify these genera whilst partly differentiating the genes correlated with each indicator. Moreover, the microbial genes and genera identified by PLS as important to explain variation in FCR and RFI were also confirmed as good predictors using LDA (prediction accuracy between 81 and 90%) for the identification of Low compared to High feed efficiency animals.

### Possible Microbial Mechanisms Detected in High Feed Efficient Beef Cattle

Possible mechanisms affecting beef cattle feed efficiency could be the result of microbial mechanisms (taxa and their metabolites) happening in the rumen or the lower gut as recent evidence suggests that both gastrointestinal sections can communicate (Steele et al., 2016). However, such interaction is not well-characterized and the exact mechanisms of gastrointestinal cross-talk in ruminants need further work.

Based on this study, several microbial mechanisms significantly more abundant in High efficient animals were identified and most of them could be involved in the rumen as well as in the lower intestine (see Table 2).

For example, the species related to *Eubacterium* were always significantly negatively correlated with RFI. *Eubacterium* is one of the dominant and major ruminal genera involved in ruminal cellulose degradation (Kozakai et al., 2007) and was found significantly more abundant in low RFI beef cattle (Elolimy et al., 2018). Similar result was described as diet dependent (concentrate) for low RFI animals (Hernandez-Sanabria et al., 2012). This genus characterized by a high diversity of potential species were confirmed carrying genes encoding for secretion system, hooking flagella and pilus formation both known to be widespread within bacteria and enhancing the capacity of the species to adhere on surface for epithelium colonization (Karuppiah et al., 2013; Green and Mecsas, 2016). In addition, some *Eubacterium* species produce butyrate and utilize acetate (Flint et al., 2007) both activities identified in high efficient animals also providing a higher energy source for the animal. As for *Eubacterium*, *Succiniclasticum*, and *Lactobacillus* are genera known to be involved in the synthesis of particular metabolites like propionate from succinate (Van Gylswyk, 1995) or lactate subsequently absorbed across the ruminal epithelium.

From the rumen microbiome, two possible mechanisms (fucose sensing and hemolysin synthesis) recognized to specifically impact the lower gut were also identified. For example, *Succiniclasticum* and *Lactobacillus* genera both significantly more abundant in low FCR animals were confirmed carrying genes involved in biofilm formation, secretion system, and more importantly fucose sensing allowing the strains to adapt to rumen and intestinal conditions (Van Gylswyk, 1995; Myer et al., 2015). For example, *Lactobacillus* genus is generally more abundant in ruminants fed with high concentrate diet (Wells et al., 1997) and will degrade fucosylated mucin recovering intestinal mucosa (but not rumen; Steele et al., 2016) using the gene K02429 as a strategy develop by the host to feed symbiotic and commensal bacteria and to regulate bacterial intestinal colonization including pathogenic colonization (Pacheco et al., 2012; Pickard and Chervonsky, 2015). However, how these mechanisms are triggered from the rumen to the intestine need further research.

In addition of adhesion genes, *Eubacterium*, the lactate producer *Bifidobacterium* and *Phenylobacterium* genera have genes encoding for hemolysin which were also associated with low RFI (K06442) and low FCR (K11068). Although hemolysin is generally produced by pathogenic bacteria for nutrient acquisition by initiating host cell lysis, it has been suggested that hemolysin can promote activation of inflammasome signals reducing pathogen colonization in the intestine (Chen et al., 2018). Results related to *Eubacterium* might be a good example of possible gastrointestinal cross-talk from bacteria detected in the rumen having a possible effect on the lower gut. More work is needed to confirm this mechanism in the rumen and the lower gut.

Therefore, our results suggest that the influences of beneficial bacteria on animal feed efficiency can be mediated by direct bacteria-cell contacts or indirectly via bacterial metabolites, such as butyrate or propionate or antipathogenic compounds from commensal bacteria.

### Possible Microbial Mechanisms Detected in Low Feed Efficient Beef Cattle

Identified mechanisms impacting host feed efficiency and located in the rumen involved two Spirochaeales genera, *Treponema* and *Sphaerochaeta.* These two genera were both positively correlated with FCR and genes associated with type IV pilus (K02652 and K02653) or secretion system (K03205). Both genera are known to inhabit the rumen with potential pathogenic activities (Smibert, 1984). Furthermore, some species within *Treponema* and *Sphaerochaeta* in the rumen showed pectinolytic activities instead of being pathogens (Xie et al., 2018) and also producing acetate, a lower energy source for the animal in comparison with butyrate and therefore potentially reducing feed efficiency (Stanton and Canale-Parola, 1980; Abt et al., 2012).

In addition to bacteria, the protozoan genus *Trichomonas* had a negative effect on feed efficiency (FCR). Generally, this genus dominates total protozoa in the rumen and the presence of protozoa compared to defaunated animals is known to reduce feed conversion efficiency in ruminants (Newbold et al., 2015). This genus was correlated with a type IV secretion system gene (K03205) one indicator of the presence of intra-ciliate Proteobacteria using T4SS to invade and survive in ruminal protozoa (Park and Yu, 2018). However, the importance of this relationship in explaining differences in feed efficiency is unclear and needs further work.

Contrasting with beneficial bacteria producing volatile fatty acids (VFA) such as butyrate or propionate, *Acidaminococcus* species are amino acid-fermenting bacteria generally located in the rumen (Cook et al., 1994). This genus through its metabolic activities in the rumen was reported to be involved in lower gut microbiome dysbiosis and gut disorder through amino acids metabolism disturbance (Lin et al., 2017). Moreover, this genus could have a contrasting impact on the physiological aspects of the host (Lin et al., 2017) like weight gain (Gough et al., 2015; Yun et al., 2017).

Other ruminal microbial species carrying genes or associated with mechanisms to colonize specifically the intestinal mucosa surface whilst avoiding the host immune system were identified and potentially having a negative impact on animal feed efficiency.

Five Proteobacteria genera were all positively correlated with high RFI. It included the motile enteric bacteria *Providencia* (K02410) which is an opportunistic pathogen in cattle normally infecting the urinary tract (Blaiotta et al., 2016). Also, the sulfate reducing bacterium *Desulfococcus* and *Variovorax* were both correlated with the T1SS gene (K02454) conferring the ability to release hemolytic toxin in a broad range of host cells (Thomas et al., 2014). Finally, *Lawsonia* was also identified and correlated with a sialic acid synthesis gene (K01654). To the best of our knowledge, this is one of the first times that *Lawsonia* known to be an obligate intracellular enteric pathogen in several animals including pig and horse (Vannucci and Gebhart, 2014) is reported in beef cattle. This genus seems to have developed a mechanism avoiding the host immune system by the production of sialic acid which is one of the main compounds constituting the mucus covering the intestine (Severi et al., 2007; Quintana-Hayashi et al., 2018). Similar mechanisms these genera may use to avoid the host immune system of the epithelia cells in the rumen. For example, Mann et al. (2018) showed that one microbial gene encoding for *N-*acetylneuraminate synthase and involved in sialic acid synthesis was highly expressed in the rumen microbiome in all cattle tested.

### Link Between Methane Emissions and Feed Efficiency in Beef Cattle

In this study, animals were fed with high concentrate diet during the finishing period to increase feed efficiency and productivity. Such diet may reduce methane emissions compared to animals receiving forage diet (Duthie et al., 2017; Cantalapiedra-Hijar et al., 2018). One of the surprising results was the lack of significant difference in methane emissions between low and high feed efficient animals whilst expected (Fitzsimons et al., 2013). Furthermore, there were no differences in CO_2_ and H_2_ emissions and VFA concentrations between both animal groups. Although the link between methane emissions and feed efficiency was not the primary aim of this study, one possible explanation for this lack of difference in methane emissions could be related to VFA metabolisms and absorption. For example, microbial metabolisms releasing of butyrate or propionate instead of acetate is known to divert H_2_ away from methanogenesis potentially reducing methane emissions and improving ruminant feed efficiency (Zhou et al., 2009; Shabat et al., 2016). However, demonstrating a higher metabolite production like for individual VFA is challenging as the rate of absorption into the blood stream is directly under the control of rumen pH as well as metabolite concentration and therefore cannot be easily determined using rumen digesta samples (Dijkstra et al., 1993). Another explanation could be due to the gap between the recording period of feed efficiency and the allocated time in the respiration chambers leading to a weak or a lack of significant correlation (Mercadante et al., 2015; Alemu et al., 2017).

This study identified both microbial genetic capacities and the microorganisms carrying the genes related and explaining a significant variability (∼40%) observed in beef cattle feed efficiency. Although knowledge about the dynamics of exchange between communities from the digesta with mucosa or between rumen digesta and lower gut is limited (Malmuthuge et al., 2015; Steele et al., 2016), it is expected that populations within the rumen digesta will compete for space and nutrients with rumen epithelia tissue-attached communities (epimural communities) also releasing metabolites and therefore impacting on the host (Pitta et al., 2016; Zhan et al., 2019). However, we acknowledge that our results are a primary step in the identification of microbial parameters impacting on host feed efficiency prior to *in vitro* or *in vivo* validation. Furthermore, other factors related to microbiome (e.g., metabolic pathways) or host responses (e.g., metabolites absorption, immune responses or behavior) are important to explain variation in host feed efficiency (Cantalapiedra-Hijar et al., 2018; Lima et al., 2019).

## Conclusion

In conclusion, microbial species carrying genes involved in adhesion and host-microbiome interaction in the rumen digesta seem to be important mechanisms explaining significant differences in animal feed efficiency but potentially sharing similar adhesion mechanisms (e.g., Type IV pilus). Based on our results, detrimental species were involved in sialic acid synthesis, and carried flagella for motility or type IV secretion system. Some others could be related to VFA and amino acid metabolisms. In contrast, beneficial bacteria, especially *Eubacterium* had the genetic capacities to form biofilms or to release hemolysin that could stimulate the immune system against pathogens in the lower gut Moreover, these species had type II secretion system and flagella for hooking and the capacity to degrade fucose as mucosal compound produced by the host intestinal epithelium.

Finally, more work is needed to better understand the dynamics and importance of exchange between microbial populations colonizing epithelial cells or the lumen during the gastrointestinal crosstalk. Such information could be used to develop molecular tools for the identification of possible probiotics or biomarkers, with the aim to improve animal production and health dietary intervention or animal breeding.

## Data Availability Statement

The datasets generated for this study can be found in the Metagenomics raw sequencing data combined with metadata of the animal experiments can be downloaded from the European Nucleotide Archive under accession PRJEB10338 and PRJEB31266.

## Ethics Statement

The animal experiment was conducted at the Beef and Sheep Research Centre of Scotland’s Rural College (SRUC, Edinburgh, United Kingdom). The experiment was approved by the Animal Experiment Committee of SRUC and was conducted in accordance with the requirements of the UK Animals (Scientific Procedures) Act 1986. Written informed consent was obtained from the owners for the participation of their animals in this study.

## Author Contributions

MA and RR conceptualized the study. MA and MW carried out the formal analysis. MA wrote the manuscript. MA, RD, C-AD, RS, MW, and RR reviewed and edited the manuscript. All authors read and approved the final manuscript.

## Conflict of Interest

The authors declare that the research was conducted in the absence of any commercial or financial relationships that could be construed as a potential conflict of interest.

## Footnotes

1 http://www.kegg.jp
