# Supplementary material for: Identification of Microbial Genetic Capacities and Potential Mechanisms Within the Rumen Microbiome Explaining Differences in Beef Cattle Feed Efficiency
Source: Front Microbiol. 2020 Jun 5;11:1229. doi: 10.3389/fmicb.2020.01229 (PMC7292206; doi:10.3389/fmicb.2020.01229)
Supplement: Supplementary file 1 [file Data_Sheet_1.docx]

**Identification of microbial genetic capacities and potential mechanisms within the rumen microbiome explaining differences in beef cattle feed efficiency.**

Marc D. Auffret*^1€^, Robert D. Stewart^2^, Richard J. Dewhurst^1^, Carol-Anne Duthie^1^, Mick Watson^2,3^, Rainer Roehe^1^*.

* Corresponding authors

[Marc.Auffret@sruc.ac.uk](mailto:Marc.Auffret@sruc.ac.uk); [Richard.Dewhurst@sruc.ac.uk](mailto:Richard.Dewhurst@sruc.ac.uk); [Carol-Anne.Duthie@sruc.ac.uk](mailto:Carol-Anne.Duthie@sruc.ac.uk); [Rob.Stewart@ed.ac.uk](mailto:Rob.Stewart@ed.ac.uk); [mick.watson@roslin.ed.ac.uk](mailto:mick.watson@roslin.ed.ac.uk); [Rainer.Roehe@sruc.ac.uk](mailto:Rainer.Roehe@sruc.ac.uk)

^1^ SRUC, Edinburgh, EH25 9RG, United Kingdom, ^2^ Division of Genetics and Genomics, The Roslin Institute and R(D)SVS, University of Edinburgh, Edinburgh, EH25 9RG, United Kingdom, ^3^ Edinburgh Genomics, The Roslin Institute and R(D)SVS, University of Edinburgh, Edinburgh, EH25 9RG, United Kingdom.

^€^Current address: Danone Nutricia Research, Innovation, Science & Nutrition Department, Gut & Microbiology Laboratories, RD 128 Avenue de la Vauve, 91767 Palaiseau Cedex, France.

**Supplementary data**

Partial Least Square (PLS) script using SAS (Version 9.1 for Windows, SAS Institute Inc., Cary, NC, USA).

Libname Pls 'C:\Users\MAuffret\Desktop\SAS PLS';

PROC IMPORT OUT= WORK.FCE

DATAFILE= "C:\Users\MAuffret\Desktop\SAS PLS\PLS input file.xlsx"

DBMS=EXCEL REPLACE;

GETNAMES=YES;

MIXED=NO;

SCANTEXT=YES;

USEDATE=YES;

SCANTIME=YES;

Run;

data Pls. ;

set work.FCE;

Run;

proc print data=Pls. ;

run;

data Pls.all_orthof3db ;

input ortho2 $1-6 ;

datalines;

###List of variables to be tested by PLS###

;

run;

title 'FCR';

ods trace on;

ods graphics on;

ods output VariableImportancePlot= vipvalues;

ods output ParmProfiles= parm_profile;

ods output CorrLoadPlot=loadvalues;

proc pls data=Pls.FCR nfac=2 plot=(ParmProfiles VIP);

class year breed;

model ###FCR or RFI### = year breed

;

run;

ods graphics off;

ods trace off;

proc print data= vipvalues ;

run;

proc print data= parm_profile;

run;

proc print data= loadvalues;

run;

**Supplementary results**

Figure S1: Variation in H_2_ (1A), CO_2_ (1B) and methane (1C) emissions (g/kg dry matter intake) between animals grouped based on feed efficiency indicators. P-value as indicator of significant difference (*P* < 0.05) between Low and High animals.

Figure S2: Variation in individual VFA concentration (mmol / mol total VFA) for acetate, propionate and butyrate (2A) or iso-butyrate, iso-valerate, valerate and branched chain fatty acids (2B) between animals grouped based on feed efficiency indicators. Acetate-to-propionate ratio was calculated and compared between Low and High animals (2C). P-value as indicator of significant difference (*P* < 0.05) between Low and High animals.

Figure S3: Canonical Variate Analysis (CVA) of the rumen microbial communities at the genus level between Low and High groups for feed efficiency. Grey diamond: High animals, Black diamond: Low animals. Circle: 95% confidence range.

Figure S4: Doughnut for the phylogenetic classification at the phylum level of MAGs-genera found significantly different between Low and High feed efficient animals by GLM.

Others include Acidobacteria, Alveolata, Amoebozoa, Aquificae, Armatimonadetes, Caldiserica, Chlamydia, Chlorobi, Chloroflexi, Chrysiogenetes, Chytridiomycota, Crenarchaeota, Cryptophyta, Deferribacteres, Deinococcus-Thermus, Desulfitobacterium, Elusimicrobia, Fibrobacteres, Fusobacteria, Gemmatimonadetes, Halobacteria, Ignavibacteriae, Kiritimatiellaeota, Microsporidia, Nitrospirae, Ochrophyta, Percolozoa, Perkinsea, Planctomycetes, Spirochaetes, Synergistetes, Tenericutes, Thaumarchaeota, Thermobaculum, Thermodesulfobacteria, Thermotogae, Verrucomicrobia. All found with less than 10 genera.

Table S1: Animal characteristics

| **Animal ID** | **Diet** | **Year** | **Breed^1^** | **FCR** | **RFI** | **Group** |
| --- | --- | --- | --- | --- | --- | --- |
| 10677WM0023 | concentrate | 2013 | Charolais | 7.698 | -1.264 | High |
| RR7 | concentrate | 2013 | Charolais | 5.848 | -1.172 | High |
| 10677WM0011 | concentrate | 2013 | Charolais | 7.350 | -1.013 | High |
| 10677WM0049 | concentrate | 2013 | Charolais | 6.160 | -0.977 | High |
| 10677WM0007 | concentrate | 2013 | Charolais | 7.464 | -0.967 | High |
| 10676WM0050 | concentrate | 2012 | Charolais | 6.760 | -0.958 | High |
| 10676WM0015 | concentrate | 2012 | Charolais | 6.442 | -0.866 | High |
| 10676WM0045 | concentrate | 2013 | Charolais | 5.764 | -0.804 | High |
| 10677WM0008 | concentrate | 2013 | Charolais | 7.270 | -0.725 | High |
| 10677WM0034 | concentrate | 2013 | Charolais | 6.845 | -0.603 | High |
| 10677WM0021 | concentrate | 2013 | Charolais | 8.306 | -0.505 | High |
| RR19 | concentrate | 2013 | Charolais | 10.288 | -0.423 | High |
| 10677WM0050 | concentrate | 2013 | Charolais | 7.142 | -0.414 | High |
| RR11 | concentrate | 2013 | Charolais | 7.001 | -0.396 | High |
| 10677WM0057 | concentrate | 2013 | Charolais | 7.818 | -0.346 | High |
| 10676WM0022 | concentrate | 2012 | Charolais | 5.938 | -0.335 | High |
| 10677WM0033 | concentrate | 2013 | Charolais | 7.550 | -0.291 | High |
| 10677WM0054 | concentrate | 2013 | Charolais | 7.594 | -0.091 | High |
| 10676WM0020 | concentrate | 2012 | Charolais | 6.718 | -0.047 | Low |
| 10678WM0028 | concentrate | 2012 | Charolais | 6.764 | -0.011 | Low |
| 10677WM0040 | concentrate | 2013 | Charolais | 6.759 | 0.011 | Low |
| 10676WM0037 | concentrate | 2012 | Charolais | 6.917 | 0.071 | Low |
| 10676WM0039 | concentrate | 2012 | Charolais | 6.858 | 0.155 | Low |
| 10676WM0026 | concentrate | 2012 | Charolais | 6.781 | 0.180 | Low |
| RR18 | concentrate | 2013 | Charolais | 7.048 | 0.215 | Low |
| 10676WM0018 | concentrate | 2012 | Charolais | 6.101 | 0.247 | Low |
| 10678WM0040 | concentrate | 2012 | Charolais | 8.026 | 0.280 | Low |
| 10678WM0036 | concentrate | 2012 | Charolais | 7.205 | 0.384 | Low |
| RR6 | concentrate | 2013 | Charolais | 9.117 | 0.437 | Low |
| 10677WM0022 | concentrate | 2013 | Charolais | 7.589 | 0.474 | Low |
| RR12 | concentrate | 2013 | Charolais | 8.567 | 0.520 | Low |
| 10678WM0038 | concentrate | 2012 | Charolais | 7.691 | 0.555 | Low |
| 10676WM0017 | concentrate | 2012 | Charolais | 7.367 | 0.612 | Low |
| 10678WM0032 | concentrate | 2012 | Charolais | 7.437 | 0.805 | Low |
| 10676WM0042 | concentrate | 2012 | Charolais | 6.970 | 0.892 | Low |
| 10678WM0042 | concentrate | 2012 | Charolais | 8.074 | 0.915 | Low |
| 10676WM0013 | concentrate | 2012 | Charolais | 7.856 | 1.576 | Low |
| RR17 | concentrate | 2013 | Luing | 11.911 | -0.458 | High |
| 10677WM0014 | concentrate | 2013 | Luing | 8.310 | -0.445 | High |
| 10676WM0016 | concentrate | 2012 | Luing | 7.810 | -0.283 | High |
| 10676WM0031 | concentrate | 2012 | Luing | 6.352 | -0.211 | High |
| 10676WM0019 | concentrate | 2012 | Luing | 5.754 | 0.147 | High |
| 10676WM0040 | concentrate | 2012 | Luing | 5.113 | 0.194 | High |
| 10678WM0031 | concentrate | 2012 | Luing | 6.634 | 0.199 | High |
| 10677WM0009 | concentrate | 2013 | Luing | 8.779 | 0.226 | High |
| 10676WM0029 | concentrate | 2012 | Luing | 6.712 | 0.295 | High |
| 10678WM0024 | concentrate | 2012 | Luing | 6.790 | 0.295 | High |
| 10676WM0047 | concentrate | 2012 | Luing | 7.552 | 0.328 | High |
| 10676WM0012 | concentrate | 2012 | Luing | 7.665 | 0.337 | High |
| 10676WM0033 | concentrate | 2012 | Luing | 6.340 | 0.420 | High |
| 10676WM0006 | concentrate | 2012 | Luing | 7.899 | 0.566 | High |
| 10676WM0027 | concentrate | 2012 | Luing | 8.635 | 0.568 | High |
| 10676WM0002 | concentrate | 2012 | Luing | 6.591 | 0.662 | High |
| 10678WM0034 | concentrate | 2012 | Luing | 6.555 | 0.671 | High |
| 10676WM0003 | concentrate | 2012 | Luing | 6.754 | 0.858 | High |
| 10676WM0032 | concentrate | 2012 | Luing | 7.605 | 0.984 | Low |
| 10676WM0005 | concentrate | 2012 | Luing | 6.504 | 1.197 | Low |
| 10678WM0041 | concentrate | 2012 | Luing | 9.779 | 1.274 | Low |
| RR8 | concentrate | 2013 | Luing | 6.966 | -1.517 | Low |
| RR20 | concentrate | 2013 | Luing | 8.096 | -0.893 | Low |
| 10677WM0038 | concentrate | 2013 | Luing | 8.248 | -0.737 | Low |
| RR5 | concentrate | 2013 | Luing | 8.568 | -0.332 | Low |
| 10677WM0051 | concentrate | 2013 | Luing | 10.020 | -0.227 | Low |
| 10677WM0025 | concentrate | 2013 | Luing | 8.083 | -0.203 | Low |
| 10677WM0010 | concentrate | 2013 | Luing | 7.293 | -0.129 | Low |
| 10677WM0035 | concentrate | 2013 | Luing | 8.131 | -0.038 | Low |
| RR14 | concentrate | 2013 | Luing | 8.030 | 0.188 | Low |
| 10677WM0012 | concentrate | 2013 | Luing | 7.004 | 0.210 | Low |
| 10677WM0056 | concentrate | 2013 | Luing | 7.987 | 0.319 | Low |
| RR13 | concentrate | 2013 | Luing | 11.538 | 0.471 | Low |
| 10677WM0015 | concentrate | 2013 | Luing | 7.878 | 1.150 | Low |
| 10677WM0055 | concentrate | 2013 | Luing | 11.613 | 1.212 | Low |

FCR: Feed Conversion Ratio; RFI: Residual Feed Intake; Group: based on FCR and RFI (Low compared to High) and balanced for Year and Breed.

^1^Crossed Charolais breed and pure Luing were used.

Table S2: Relative abundance of microbial genera significantly different (*P* < 0.05) between Low and High groups.

| **Domain** | **Phylum** | **Genus-MAG** | **Mean Low** | **Mean High** | ***F-*value** | ***P-*value** |
| --- | --- | --- | --- | --- | --- | --- |
| Bacteria | Synergistetes | *Acetomicrobium* | 0.0015 | 0.0014 | 3.486 | 0.012 |
| Bacteria | Tenericutes | *Acholeplasma* | 0.0143 | 0.0155 | 6.039 | 0.000 |
| Bacteria | Firmicutes | *Acidaminococcus* | 0.9713 | 1.0510 | 5.514 | 0.001 |
| Bacteria | Proteobacteria | *Acidihalobacter* | 0.0181 | 0.0187 | 4.740 | 0.002 |
| Bacteria | Actinobacteria | *Actinomyces* | 0.0635 | 0.0621 | 3.670 | 0.009 |
| Bacteria | Actinobacteria | *Actinoplanes* | 0.0226 | 0.0222 | 6.707 | 0.000 |
| Bacteria | Actinobacteria | *Actinosynnema* | 0.0032 | 0.0031 | 2.044 | 0.098 |
| Bacteria | Actinobacteria | *Adlercreutzia* | 0.0658 | 0.0580 | 7.289 | 0.000 |
| Bacteria | Bacteroidetes | *Aequorivita* | 0.0022 | 0.0024 | 5.619 | 0.001 |
| Bacteria | Proteobacteria | *Agrobacterium* | 0.0386 | 0.0383 | 6.594 | 0.000 |
| Bacteria | Proteobacteria | *Alcaligenes* | 0.0028 | 0.0025 | 3.405 | 0.014 |
| Bacteria | Proteobacteria | *Alcanivorax* | 0.0198 | 0.0218 | 7.046 | 0.000 |
| Bacteria | Bacteroidetes | *Algibacter* | 0.0038 | 0.0040 | 3.513 | 0.012 |
| Bacteria | Bacteroidetes | *Algoriphagus* | 0.0032 | 0.0034 | 6.656 | 0.000 |
| Bacteria | Firmicutes | *Alicyclobacillus* | 0.0149 | 0.0148 | 3.196 | 0.018 |
| Bacteria | Bacteroidetes | *Alistipes* | 0.2031 | 0.2129 | 2.475 | 0.053 |
| Bacteria | Firmicutes | *Allisonella* | 0.0413 | 0.0445 | 8.047 | 0.000 |
| Bacteria | Proteobacteria | *Allofrancisella* | 0.0176 | 0.0179 | 4.653 | 0.002 |
| Bacteria | Proteobacteria | *Altererythrobacter* | 0.0146 | 0.0158 | 7.353 | 0.000 |
| Bacteria | Synergistetes | *Aminobacterium* | 0.0123 | 0.0125 | 5.245 | 0.001 |
| Bacteria | Synergistetes | *Aminomonas* | 0.0042 | 0.0041 | 4.031 | 0.006 |
| Bacteria | Firmicutes | *Ammonifex* | 0.0031 | 0.0029 | 7.156 | 0.000 |
| Bacteria | Actinobacteria | *Amycolatopsis* | 0.0247 | 0.0453 | 2.740 | 0.036 |
| Bacteria | Firmicutes | *Anaerococcus* | 0.0026 | 0.0027 | 4.097 | 0.005 |
| Bacteria | Chloroflexi | *Anaerolinea* | 0.0461 | 0.0462 | 3.509 | 0.012 |
| Bacteria | Proteobacteria | *Anaeromyxobacter* | 0.1048 | 0.1221 | 3.602 | 0.010 |
| Bacteria | Firmicutes | *Anoxybacillus* | 0.0031 | 0.0035 | 2.051 | 0.097 |
| Bacteria | Bacteroidetes | *Arachidicoccus* | 0.0020 | 0.0019 | 5.677 | 0.001 |
| Bacteria | Actinobacteria | *Arcanobacterium* | 0.0122 | 0.0119 | 7.413 | 0.000 |
| Bacteria | Proteobacteria | *Archangium* | 0.0142 | 0.0162 | 3.562 | 0.011 |
| Bacteria | Proteobacteria | *Arsenophonus* | 0.0520 | 0.0532 | 5.771 | 0.000 |
| Bacteria | Proteobacteria | *Asticcacaulis* | 0.0102 | 0.0087 | 8.229 | 0.000 |
| Fungi | Basidiomycota | *Auricularia* | 0.0039 | 0.0034 | 3.693 | 0.009 |
| Bacteria | Proteobacteria | *Azoarcus* | 0.0314 | 0.0319 | 2.157 | 0.084 |
| Bacteria | Proteobacteria | *Azorhizobium* | 0.0126 | 0.0122 | 3.922 | 0.006 |
| Bacteria | Proteobacteria | *Azospira* | 0.0137 | 0.0139 | 3.555 | 0.011 |
| Bacteria | Proteobacteria | *Azotobacter* | 0.0125 | 0.0129 | 2.061 | 0.096 |
| Bacteria | Firmicutes | *Bacillus* | 0.1397 | 0.1586 | 5.795 | 0.000 |
| Bacteria | Proteobacteria | *Bartonella* | 0.0042 | 0.0045 | 3.705 | 0.009 |
| Bacteria | Proteobacteria | *Basfia* | 0.0025 | 0.0020 | 2.566 | 0.046 |
| Bacteria | Actinobacteria | *Bifidobacterium* | 0.9424 | 0.3718 | 3.526 | 0.011 |
| Bacteria | Proteobacteria | *Blastomonas* | 0.0046 | 0.0046 | 3.600 | 0.010 |
| Bacteria | Proteobacteria | *Bordetella* | 0.0641 | 0.0637 | 3.419 | 0.013 |
| Fungi | Ascomycota | *Botrytis* | 0.0104 | 0.0128 | 3.205 | 0.018 |
| Bacteria | Firmicutes | *Brevibacillus* | 0.0044 | 0.0043 | 5.973 | 0.000 |
| Bacteria | Actinobacteria | *Brevibacterium* | 0.0086 | 0.0092 | 2.562 | 0.046 |
| Bacteria | Proteobacteria | *Brevundimonas* | 0.0204 | 0.0214 | 3.824 | 0.007 |
| Bacteria | Proteobacteria | *Brucella* | 0.0045 | 0.0036 | 8.041 | 0.000 |
| Bacteria | Firmicutes | *Caldanaerobacter* | 0.0023 | 0.0020 | 5.180 | 0.001 |
| Bacteria | Firmicutes | *Caldicellulosiruptor* | 0.0118 | 0.0132 | 3.491 | 0.012 |
| Bacteria | Thermodesulfobacteria | *Caldimicrobium* | 0.0012 | 0.0015 | 2.084 | 0.093 |
| Bacteria | Caldiserica | *Caldisericum* | 0.0010 | 0.0014 | 9.254 | 0.000 |
| Bacteria | Cyanobacteria | *Calothrix* | 0.5007 | 1.0033 | 2.983 | 0.025 |
| Bacteria | Proteobacteria | *Campylobacter* | 0.0449 | 0.0366 | 3.919 | 0.006 |
| Bacteria | Bacteroidetes | Candidatus *Azobacteroides* | 0.0040 | 0.0049 | 3.240 | 0.017 |
| Bacteria | Proteobacteria | *Candidatus Babela* | 0.0013 | 0.0017 | 3.236 | 0.017 |
| Archaea | Euryarchaeota | Candidatus *Halobonum* | 0.0021 | 0.0017 | 3.616 | 0.010 |
| Bacteria | Tenericutes | Candidatus *Izimaplasma* | 0.0095 | 0.0093 | 3.481 | 0.012 |
| Bacteria | Proteobacteria | Candidatus *Liberibacter* | 0.1161 | 0.1732 | 3.770 | 0.008 |
| Bacteria | Proteobacteria | Candidatus *Paracaedibacter* | 0.0028 | 0.0031 | 2.117 | 0.088 |
| Bacteria | Proteobacteria | Candidatus *Pelagibacter* | 0.0023 | 0.0025 | 4.734 | 0.002 |
| Bacteria | Tenericutes | Candidatus *Phytoplasma* | 0.0032 | 0.0047 | 2.580 | 0.045 |
| Bacteria | Chlamydiae | Candidatus *Protochlamydia* | 0.0026 | 0.0028 | 3.519 | 0.012 |
| Bacteria | Bacteroidetes | Candidatus *Sulcia* | 0.0022 | 0.0023 | 4.109 | 0.005 |
| Bacteria | Bacteroidetes | *Capnocytophaga* | 0.0283 | 0.0257 | 3.423 | 0.013 |
| Bacteria | Proteobacteria | *Castellaniella* | 0.0119 | 0.0122 | 9.016 | 0.000 |
| Bacteria | Proteobacteria | *Caulobacter* | 0.0226 | 0.0213 | 2.231 | 0.075 |
| Bacteria | Proteobacteria | *Cedecea* | 0.0096 | 0.0106 | 5.417 | 0.001 |
| Bacteria | Firmicutes | *Cellulosilyticum* | 0.0103 | 0.0117 | 4.351 | 0.003 |
| Bacteria | Proteobacteria | *Cellvibrio* | 0.0031 | 0.0031 | 8.853 | 0.000 |
| Bacteria | Proteobacteria | *Chelatococcus* | 0.0192 | 0.0200 | 2.525 | 0.049 |
| Bacteria | Bacteroidetes | *Chitinophaga* | 0.0099 | 0.0097 | 2.306 | 0.067 |
| Bacteria | Chlorobi | *Chlorobium* | 0.0197 | 0.0213 | 4.601 | 0.002 |
| Bacteria | Proteobacteria | *Chromohalobacter* | 0.0052 | 0.0049 | 3.317 | 0.015 |
| Bacteria | Armatimonadetes | *Chthonomonas* | 0.0037 | 0.0040 | 7.853 | 0.000 |
| Bacteria | Proteobacteria | *Citrobacter* | 0.0267 | 0.0267 | 3.580 | 0.011 |
| Fungi | Ascomycota | *Cladophialophora* | 0.0081 | 0.0110 | 4.916 | 0.002 |
| Bacteria | Actinobacteria | *Clavibacter* | 0.0190 | 0.0189 | 2.132 | 0.087 |
| Bacteria | Synergistetes | *Cloacibacillus* | 0.0199 | 0.0202 | 4.245 | 0.004 |
| Bacteria | Firmicutes | *Clostridium* | 0.6274 | 0.6065 | 4.686 | 0.002 |
| Bacteria | Proteobacteria | *Comamonas* | 0.0194 | 0.0210 | 4.437 | 0.003 |
| Bacteria | Actinobacteria | *Conexibacter* | 0.0108 | 0.0109 | 4.611 | 0.002 |
| Bacteria | Proteobacteria | *Confluentimicrobium* | 0.0035 | 0.0035 | 2.260 | 0.072 |
| Bacteria | Proteobacteria | *Congregibacter* | 0.0038 | 0.0043 | 5.717 | 0.001 |
| Bacteria | Verrucomicrobia | *Coraliomargarita* | 0.0048 | 0.0053 | 10.913 | 0.000 |
| Bacteria | Actinobacteria | *Coriobacterium* | 0.0218 | 0.0190 | 9.001 | 0.000 |
| Bacteria | Actinobacteria | *Corynebacterium* | 0.1937 | 0.1892 | 5.044 | 0.001 |
| Bacteria | Proteobacteria | *Coxiella* | 0.0010 | 0.0014 | 2.728 | 0.036 |
| Bacteria | Proteobacteria | *Croceicoccus* | 0.0028 | 0.0030 | 3.918 | 0.006 |
| Fungi | Basidiomycota | *Cryptococcus* | 0.0032 | 0.0044 | 2.247 | 0.073 |
| Bacteria | Proteobacteria | *Cupriavidus* | 0.0441 | 0.0451 | 3.815 | 0.008 |
| Bacteria | Actinobacteria | *Curtobacterium* | 0.0102 | 0.0089 | 2.677 | 0.039 |
| Bacteria | Thermotogae | *Defluviitoga* | 0.0011 | 0.0012 | 3.375 | 0.014 |
| Bacteria | Deinococcus-Thermus | *Deinococcus* | 0.0609 | 0.0605 | 2.359 | 0.062 |
| Bacteria | Actinobacteria | *Denitrobacterium* | 0.0783 | 0.0671 | 2.488 | 0.052 |
| Bacteria | Deferribacteres | *Denitrovibrio* | 0.0020 | 0.0022 | 7.559 | 0.000 |
| Bacteria | Actinobacteria | *Dermacoccus* | 0.0037 | 0.0035 | 6.242 | 0.000 |
| Bacteria | Desulfitobacterium | *Desulfarculus* | 0.0093 | 0.0099 | 2.298 | 0.068 |
| Bacteria | Firmicutes | *Desulfitobacterium* | 0.0175 | 0.0178 | 5.434 | 0.001 |
| Bacteria | Proteobacteria | *Desulfobacter* | 0.0025 | 0.0027 | 3.419 | 0.013 |
| Bacteria | Proteobacteria | *Desulfobacterium* | 0.0032 | 0.0033 | 10.354 | 0.000 |
| Bacteria | Proteobacteria | *Desulfobacula* | 0.0022 | 0.0029 | 3.829 | 0.007 |
| Bacteria | Proteobacteria | *Desulfobulbus* | 0.0119 | 0.0120 | 3.490 | 0.012 |
| Bacteria | Proteobacteria | *Desulfocapsa* | 0.0018 | 0.0017 | 5.369 | 0.001 |
| Bacteria | Proteobacteria | *Desulfococcus* | 0.0198 | 0.0216 | 2.908 | 0.028 |
| Bacteria | Proteobacteria | *Desulfohalobium* | 0.0042 | 0.0047 | 3.065 | 0.022 |
| Bacteria | Proteobacteria | *Desulfomicrobium* | 0.0184 | 0.0199 | 6.179 | 0.000 |
| Bacteria | Proteobacteria | *Desulfomonile* | 0.0022 | 0.0023 | 7.562 | 0.000 |
| Bacteria | Firmicutes | *Desulfosporosinus* | 0.0110 | 0.0122 | 4.823 | 0.002 |
| Bacteria | Proteobacteria | *Desulfovibrio* | 0.2143 | 0.2186 | 3.446 | 0.013 |
| Bacteria | Chrysiogenetes | *Desulfurispirillum* | 0.0114 | 0.0130 | 2.519 | 0.049 |
| Bacteria | Aquificae | *Desulfurobacterium* | 0.0014 | 0.0015 | 4.921 | 0.002 |
| Bacteria | Firmicutes | *Dialister* | 0.0253 | 0.0277 | 9.494 | 0.000 |
| Fungi | Basidiomycota | *Dichomitus* | 0.0009 | 0.0011 | 5.993 | 0.000 |
| Bacteria | Proteobacteria | *Dinoroseobacter* | 0.0030 | 0.0029 | 7.269 | 0.000 |
| Bacteria | Bacteroidetes | *Dokdonia* | 0.0036 | 0.0039 | 3.473 | 0.012 |
| Bacteria | Proteobacteria | *Edwardsiella* | 0.0375 | 0.0386 | 2.418 | 0.057 |
| Bacteria | Actinobacteria | *Eggerthella* | 0.0828 | 0.0720 | 6.768 | 0.000 |
| Bacteria | Proteobacteria | *Ehrlichia* | 0.0034 | 0.0035 | 6.492 | 0.000 |
| Protist | Alveolata | *Eimeria* | 0.8826 | 1.1673 | 2.616 | 0.043 |
| Bacteria | Bacteroidetes | *Elizabethkingia* | 0.0047 | 0.0049 | 5.087 | 0.001 |
| Bacteria | Elusimicrobia | *Elusimicrobium* | 0.0024 | 0.0035 | 3.507 | 0.012 |
| Fungi | Ascomycota | *Endocarpon* | 0.0024 | 0.0027 | 6.406 | 0.000 |
| Bacteria | Elusimicrobia | *Endomicrobium* | 0.0029 | 0.0033 | 3.518 | 0.012 |
| Protist | Amoebozoa | *Entamoeba* | 0.0727 | 0.1207 | 2.462 | 0.054 |
| Bacteria | Firmicutes | *Enterococcus* | 0.0299 | 0.0324 | 8.355 | 0.000 |
| Fungi | Ascomycota | *Eremothecium* | 0.0040 | 0.0061 | 2.848 | 0.031 |
| Bacteria | Proteobacteria | *Erwinia* | 0.0252 | 0.0256 | 2.485 | 0.052 |
| Bacteria | Proteobacteria | *Escherichia* | 0.0239 | 0.0237 | 2.617 | 0.043 |
| Bacteria | Firmicutes | *Ethanoligenens* | 0.0522 | 0.0531 | 4.477 | 0.003 |
| Bacteria | Firmicutes | *Eubacterium* | 1.5236 | 1.4375 | 10.994 | 0.000 |
| Bacteria | Firmicutes | *Exiguobacterium* | 0.0113 | 0.0103 | 2.223 | 0.076 |
| Fungi | Ascomycota | *Exophiala* | 0.0138 | 0.0147 | 8.754 | 0.000 |
| Bacteria | Firmicutes | *Ezakiella* | 0.0037 | 0.0038 | 4.241 | 0.004 |
| Bacteria | Firmicutes | *Faecalibaculum* | 0.0186 | 0.0181 | 2.639 | 0.041 |
| Bacteria | Firmicutes | *Faecalitalea* | 0.0167 | 0.0154 | 4.281 | 0.004 |
| Bacteria | Thermotogae | *Fervidobacterium* | 0.0016 | 0.0018 | 2.203 | 0.078 |
| Bacteria | Fibrobacteres | *Fibrobacter* | 0.4766 | 0.6918 | 3.438 | 0.013 |
| Fungi | Basidiomycota | *Fibroporia* | 0.0010 | 0.0011 | 4.018 | 0.006 |
| Bacteria | Firmicutes | *Fictibacillus* | 0.0034 | 0.0037 | 2.208 | 0.078 |
| Bacteria | Armatimonadetes | *Fimbriimonas* | 0.0036 | 0.0039 | 3.896 | 0.007 |
| Bacteria | Firmicutes | *Finegoldia* | 0.0106 | 0.0116 | 4.766 | 0.002 |
| Bacteria | Bacteroidetes | *Flavobacterium* | 0.0286 | 0.0298 | 3.210 | 0.018 |
| Bacteria | Bacteroidetes | *Fluviicola* | 0.0029 | 0.0033 | 3.003 | 0.024 |
| Fungi | Basidiomycota | *Fomitiporia* | 0.0010 | 0.0018 | 2.319 | 0.066 |
| Bacteria | Bacteroidetes | *Formosa* | 0.0039 | 0.0041 | 8.489 | 0.000 |
| Bacteria | Proteobacteria | *Francisella* | 0.0238 | 0.0261 | 2.783 | 0.034 |
| Bacteria | Actinobacteria | *Frankia* | 0.0218 | 0.0201 | 9.124 | 0.000 |
| Bacteria | Proteobacteria | *Frateuria* | 0.0047 | 0.0054 | 3.089 | 0.022 |
| Fungi | Ascomycota | *Fusarium* | 0.0147 | 0.0186 | 3.195 | 0.018 |
| Bacteria | Fusobacteria | *Fusobacterium* | 0.0288 | 0.0322 | 2.876 | 0.029 |
| Bacteria | Proteobacteria | *Gallibacterium* | 0.0028 | 0.0025 | 9.737 | 0.000 |
| Bacteria | Actinobacteria | *Gardnerella* | 0.0043 | 0.0052 | 2.412 | 0.058 |
| Bacteria | Cyanobacteria | *Geitlerinema* | 0.0032 | 0.0036 | 6.398 | 0.000 |
| Bacteria | Firmicutes | *Gemella* | 0.0026 | 0.0031 | 4.510 | 0.003 |
| Bacteria | Gemmatimonadetes | *Gemmatimonas* | 0.0098 | 0.0107 | 10.670 | 0.000 |
| Bacteria | Actinobacteria | *Geodermatophilus* | 0.0035 | 0.0033 | 5.748 | 0.000 |
| Archaea | Euryarchaeota | *Geoglobus* | 0.0013 | 0.0009 | 4.925 | 0.002 |
| Bacteria | Proteobacteria | *Gilliamella* | 0.0029 | 0.0035 | 2.732 | 0.036 |
| Bacteria | Proteobacteria | *Glaciecola* | 0.0011 | 0.0014 | 2.766 | 0.034 |
| Fungi | Ascomycota | *Glarea* | 0.0034 | 0.0037 | 5.630 | 0.001 |
| Bacteria | Cyanobacteria | *Gloeobacter* | 0.0152 | 0.0151 | 5.293 | 0.001 |
| Bacteria | Actinobacteria | *Gordonia* | 0.0285 | 0.0283 | 2.789 | 0.033 |
| Bacteria | Actinobacteria | *Gordonibacter* | 0.0781 | 0.0643 | 6.550 | 0.000 |
| Bacteria | Proteobacteria | *Granulibacter* | 0.0048 | 0.0048 | 4.718 | 0.002 |
| Protist | Alveolata | *Gregarina* | 0.0031 | 0.0042 | 5.344 | 0.001 |
| Euk | Cryptophyta | *Guillardia* | 0.0216 | 0.0283 | 2.753 | 0.035 |
| Bacteria | Proteobacteria | *Gynuella* | 0.0023 | 0.0022 | 2.346 | 0.064 |
| Bacteria | Proteobacteria | *Haematospirillum* | 0.0038 | 0.0043 | 7.153 | 0.000 |
| Bacteria | Proteobacteria | *Hafnia* | 0.0048 | 0.0046 | 4.305 | 0.004 |
| Archaea | Euryarchaeota | *Haladaptatus* | 0.0019 | 0.0019 | 9.430 | 0.000 |
| Archaea | Euryarchaeota | *Halalkalicoccus* | 0.0020 | 0.0022 | 6.811 | 0.000 |
| Bacteria | Proteobacteria | *Haliangium* | 0.0127 | 0.0111 | 5.090 | 0.001 |
| Archaea | Euryarchaeota | *Halobacterium* | 0.0045 | 0.0046 | 6.076 | 0.000 |
| Archaea | Euryarchaeota | *Halogeometricum* | 0.0017 | 0.0019 | 2.451 | 0.055 |
| Archaea | Euryarchaeota | *Halogranum* | 0.0025 | 0.0027 | 2.845 | 0.031 |
| Archaea | Euryarchaeota | *Halomicrobium* | 0.0024 | 0.0026 | 5.628 | 0.001 |
| Bacteria | Proteobacteria | *Halomonas* | 0.0434 | 0.0474 | 4.844 | 0.002 |
| Archaea | Euryarchaeota | *Halopenitus* | 0.0031 | 0.0027 | 4.415 | 0.003 |
| Archaea | Euryarchaeota | *Halopiger* | 0.0018 | 0.0020 | 6.652 | 0.000 |
| Archaea | Euryarchaeota | *Haloquadratum* | 0.0018 | 0.0013 | 2.294 | 0.068 |
| Archaea | Euryarchaeota | *Halosimplex* | 0.0031 | 0.0034 | 7.688 | 0.000 |
| Bacteria | Firmicutes | *Halothermothrix* | 0.0017 | 0.0018 | 2.873 | 0.029 |
| Bacteria | Proteobacteria | *Herbaspirillum* | 0.0199 | 0.0212 | 2.920 | 0.028 |
| Fungi | Basidiomycota | *Heterobasidion* | 0.0035 | 0.0038 | 7.209 | 0.000 |
| Bacteria | Actinobacteria | *Hoyosella* | 0.0016 | 0.0014 | 2.437 | 0.056 |
| Bacteria | Proteobacteria | *Hydrogenophaga* | 0.0180 | 0.0190 | 2.620 | 0.043 |
| Bacteria | Bacteroidetes | *Hymenobacter* | 0.1487 | 0.1628 | 4.288 | 0.004 |
| Bacteria | Proteobacteria | *Hyphomicrobium* | 0.0112 | 0.0113 | 7.877 | 0.000 |
| Bacteria | Proteobacteria | *Idiomarina* | 0.0013 | 0.0020 | 5.313 | 0.001 |
| Archaea | Crenarchaeota | *Ignicoccus* | 0.0013 | 0.0014 | 6.213 | 0.000 |
| Bacteria | Actinobacteria | *Ilumatobacter* | 0.0047 | 0.0046 | 6.255 | 0.000 |
| Bacteria | Actinobacteria | *Intrasporangium* | 0.0034 | 0.0031 | 4.426 | 0.003 |
| Fungi | Ascomycota | *Isaria* | 0.0584 | 0.1158 | 2.681 | 0.039 |
| Bacteria | Firmicutes | *Jeotgalibaca* | 0.0022 | 0.0019 | 4.346 | 0.004 |
| Bacteria | Firmicutes | *Jeotgalibacillus* | 0.0022 | 0.0023 | 3.029 | 0.023 |
| Bacteria | Firmicutes | *Jeotgalicoccus* | 0.0023 | 0.0024 | 3.567 | 0.011 |
| Bacteria | Synergistetes | *Jonquetella* | 0.0116 | 0.0121 | 2.270 | 0.071 |
| Fungi | Basidiomycota | *Kalmanozyma* | 0.0029 | 0.0026 | 9.051 | 0.000 |
| Bacteria | Firmicutes | *Kandleria* | 0.0946 | 0.0863 | 2.744 | 0.036 |
| Fungi | Ascomycota | *Kazachstania* | 0.0020 | 0.0034 | 2.638 | 0.042 |
| Bacteria | Actinobacteria | *Kibdelosporangium* | 0.0032 | 0.0031 | 3.006 | 0.024 |
| Bacteria | Actinobacteria | *Kineococcus* | 0.0037 | 0.0039 | 3.578 | 0.011 |
| Bacteria | Proteobacteria | *Kinetoplastibacterium* | 0.0032 | 0.0040 | 5.818 | 0.000 |
| Bacteria | Kiritimatiellaeota | *Kiritimatiella* | 0.0091 | 0.0094 | 3.313 | 0.016 |
| Bacteria | Actinobacteria | *Kitasatospora* | 0.0039 | 0.0033 | 2.475 | 0.053 |
| Bacteria | Proteobacteria | *Kluyvera* | 0.0042 | 0.0046 | 4.859 | 0.002 |
| Bacteria | Firmicutes | *Kurthia* | 0.0023 | 0.0026 | 3.681 | 0.009 |
| Bacteria | Actinobacteria | *Kytococcus* | 0.0046 | 0.0039 | 6.731 | 0.000 |
| Bacteria | Proteobacteria | *Labrenzia* | 0.0087 | 0.0093 | 3.223 | 0.018 |
| Bacteria | Proteobacteria | *Lacimicrobium* | 0.0037 | 0.0039 | 7.804 | 0.000 |
| Bacteria | Bacteroidetes | *Lacinutrix* | 0.0036 | 0.0034 | 4.094 | 0.005 |
| Bacteria | Firmicutes | *Lactobacillus* | 0.4167 | 0.4048 | 2.744 | 0.036 |
| Bacteria | Firmicutes | *Lactococcus* | 0.0090 | 0.0092 | 8.569 | 0.000 |
| Bacteria | Proteobacteria | *Lawsonia* | 0.0017 | 0.0026 | 3.237 | 0.017 |
| Bacteria | Bacteroidetes | *Leadbetterella* | 0.0030 | 0.0036 | 10.726 | 0.000 |
| Bacteria | Proteobacteria | *Lelliottia* | 0.0027 | 0.0030 | 2.620 | 0.043 |
| Bacteria | Cyanobacteria | *Leptolyngbya* | 0.0099 | 0.0129 | 4.004 | 0.006 |
| Bacteria | Nitrospirae | *Leptospirillum* | 0.0022 | 0.0020 | 3.113 | 0.021 |
| Bacteria | Proteobacteria | *Leptothrix* | 0.0104 | 0.0110 | 6.246 | 0.000 |
| Bacteria | Fusobacteria | *Leptotrichia* | 0.0111 | 0.0112 | 2.446 | 0.055 |
| Bacteria | Actinobacteria | *Libanicoccus* | 0.1544 | 0.1236 | 6.005 | 0.000 |
| Bacteria | Firmicutes | *Listeria* | 0.0119 | 0.0116 | 6.107 | 0.000 |
| Fungi | Ascomycota | *Lodderomyces* | 0.0257 | 0.0386 | 2.166 | 0.082 |
| Bacteria | Actinobacteria | *Luteipulveratus* | 0.0048 | 0.0045 | 4.326 | 0.004 |
| Bacteria | Firmicutes | *Macrococcus* | 0.0016 | 0.0021 | 3.055 | 0.023 |
| Bacteria | Firmicutes | *Mageeibacillus* | 0.0117 | 0.0115 | 7.849 | 0.000 |
| Bacteria | Proteobacteria | *Magnetospira* | 0.0036 | 0.0039 | 3.326 | 0.015 |
| Bacteria | Proteobacteria | *Magnetospirillum* | 0.0173 | 0.0168 | 2.743 | 0.036 |
| Bacteria | Firmicutes | *Mahella* | 0.0026 | 0.0025 | 3.159 | 0.019 |
| Bacteria | Proteobacteria | *Maricaulis* | 0.0035 | 0.0032 | 2.791 | 0.033 |
| Bacteria | Firmicutes | *Marinilactibacillus* | 0.0027 | 0.0028 | 4.445 | 0.003 |
| Bacteria | Deinococcus-Thermus | *Marinithermus* | 0.0043 | 0.0050 | 4.782 | 0.002 |
| Bacteria | Thermotogae | *Marinitoga* | 0.0042 | 0.0044 | 5.148 | 0.001 |
| Bacteria | Proteobacteria | *Marinovum* | 0.0043 | 0.0039 | 4.587 | 0.002 |
| Bacteria | Proteobacteria | *Martelella* | 0.0121 | 0.0130 | 7.816 | 0.000 |
| Bacteria | Firmicutes | *Megamonas* | 0.0094 | 0.0100 | 3.222 | 0.018 |
| Bacteria | Firmicutes | *Megasphaera* | 0.2179 | 0.1894 | 2.584 | 0.045 |
| Bacteria | Ignavibacteriae | *Melioribacter* | 0.0017 | 0.0018 | 5.759 | 0.000 |
| Bacteria | Firmicutes | *Melissococcus* | 0.0025 | 0.0028 | 5.255 | 0.001 |
| Bacteria | Tenericutes | *Mesoplasma* | 0.0025 | 0.0028 | 4.402 | 0.003 |
| Fungi | Ascomycota | *Metarhizium* | 0.0178 | 0.0202 | 2.439 | 0.056 |
| Archaea | Euryarchaeota | *Methanobacterium* | 0.0417 | 0.0345 | 2.367 | 0.062 |
| Archaea | Euryarchaeota | *Methanocaldococcus* | 0.0035 | 0.0044 | 2.131 | 0.087 |
| Archaea | Euryarchaeota | *Methanocella* | 0.0024 | 0.0023 | 3.511 | 0.012 |
| Archaea | Euryarchaeota | *Methanofollis* | 0.0045 | 0.0042 | 2.460 | 0.054 |
| Archaea | Euryarchaeota | *Methanolacinia* | 0.0016 | 0.0012 | 3.870 | 0.007 |
| Archaea | Euryarchaeota | *Methanomassiliicoccus* | 0.0046 | 0.0045 | 10.063 | 0.000 |
| Archaea | Euryarchaeota | *Methanoregula* | 0.0031 | 0.0034 | 10.105 | 0.000 |
| Archaea | Euryarchaeota | *Methanosaeta* | 0.0035 | 0.0038 | 6.481 | 0.000 |
| Archaea | Euryarchaeota | *Methanosphaera* | 0.0187 | 0.0193 | 7.262 | 0.000 |
| Bacteria | Proteobacteria | *Methylobacillus* | 0.0018 | 0.0016 | 2.905 | 0.028 |
| Bacteria | Proteobacteria | *Methylomicrobium* | 0.0100 | 0.0105 | 12.153 | 0.000 |
| Bacteria | Proteobacteria | *Methylophaga* | 0.0019 | 0.0024 | 2.607 | 0.043 |
| Bacteria | Proteobacteria | *Methylophilus* | 0.0026 | 0.0027 | 4.123 | 0.005 |
| Bacteria | Proteobacteria | *Methylovorus* | 0.0036 | 0.0037 | 2.980 | 0.025 |
| Bacteria | Proteobacteria | *Microbulbifer* | 0.0238 | 0.0255 | 3.188 | 0.019 |
| Bacteria | Cyanobacteria | *Microcoleus* | 0.0021 | 0.0030 | 2.331 | 0.065 |
| Fungi | Microsporida | *Mitosporidium* | 0.0033 | 0.0053 | 2.670 | 0.040 |
| Bacteria | Firmicutes | *Mitsuokella* | 0.5389 | 0.5265 | 5.449 | 0.001 |
| Bacteria | Actinobacteria | *Mobiluncus* | 0.0013 | 0.0017 | 3.734 | 0.008 |
| Bacteria | Actinobacteria | *Modestobacter* | 0.0103 | 0.0106 | 4.358 | 0.003 |
| Bacteria | Firmicutes | *Murdochiella* | 0.0151 | 0.0163 | 4.242 | 0.004 |
| Bacteria | Bacteroidetes | *Muricauda* | 0.0087 | 0.0097 | 5.719 | 0.001 |
| Bacteria | Actinobacteria | *Mycobacterium* | 0.1134 | 0.1097 | 2.076 | 0.094 |
| Bacteria | Bacteroidetes | *Myroides* | 0.0097 | 0.0100 | 3.132 | 0.020 |
| Bacteria | Proteobacteria | *Myxococcus* | 0.0426 | 0.0410 | 3.933 | 0.006 |
| Protist | Percolozoa | *Naegleria* | 0.0202 | 0.0335 | 2.250 | 0.073 |
| Protist | Ochrophyta | *Nannochloropsis* | 0.2087 | 0.1618 | 2.642 | 0.041 |
| Bacteria | Firmicutes | *Natranaerobius* | 0.0020 | 0.0021 | 8.758 | 0.000 |
| Archaea | Halobacteria | *Natronococcus* | 0.0016 | 0.0015 | 3.640 | 0.010 |
| Archaea | Halobacteria | *Natronomonas* | 0.0019 | 0.0018 | 3.250 | 0.017 |
| Archaea | Halobacteria | *Natronorubrum* | 0.0025 | 0.0028 | 6.433 | 0.000 |
| Fungi | Ascomycota | *Naumovozyma* | 0.0074 | 0.0115 | 2.278 | 0.070 |
| Bacteria | Firmicutes | *Negativicoccus* | 0.7746 | 0.7820 | 2.052 | 0.097 |
| Bacteria | Actinobacteria | *Neomicrococcus* | 0.0027 | 0.0025 | 2.468 | 0.053 |
| Bacteria | Proteobacteria | *Neorickettsia* | 0.0013 | 0.0012 | 10.436 | 0.000 |
| Protist | Alveolata | *Neospora* | 0.0041 | 0.0057 | 6.478 | 0.000 |
| Bacteria | Bacteroidetes | *Niabella* | 0.0099 | 0.0090 | 4.299 | 0.004 |
| Bacteria | Proteobacteria | *Nitratireductor* | 0.0044 | 0.0048 | 3.152 | 0.020 |
| Bacteria | Proteobacteria | *Nitratiruptor* | 0.0023 | 0.0042 | 4.339 | 0.004 |
| Bacteria | Proteobacteria | *Nitrosococcus* | 0.0043 | 0.0044 | 2.134 | 0.086 |
| Archaea | Thaumarchaeota | *Nitrosopumilus* | 0.0016 | 0.0016 | 2.126 | 0.087 |
| Bacteria | Actinobacteria | *Nocardia* | 0.0162 | 0.0150 | 2.539 | 0.048 |
| Bacteria | Actinobacteria | *Nocardiopsis* | 0.0090 | 0.0095 | 4.061 | 0.005 |
| Bacteria | Cyanobacteria | *Nodularia* | 0.0744 | 0.1473 | 2.938 | 0.027 |
| Fungi | Other | *Nosema* | 0.0030 | 0.0052 | 2.903 | 0.028 |
| Bacteria | Cyanobacteria | *Nostoc* | 0.8625 | 1.7009 | 3.033 | 0.023 |
| Bacteria | Proteobacteria | *Novosphingobium* | 0.0153 | 0.0157 | 3.645 | 0.010 |
| Bacteria | Firmicutes | *Oceanobacillus* | 0.0051 | 0.0048 | 7.145 | 0.000 |
| Bacteria | Firmicutes | *Oenococcus* | 0.0019 | 0.0020 | 5.376 | 0.001 |
| Fungi | Ascomycota | *Ogataea* | 0.0015 | 0.0018 | 4.047 | 0.005 |
| Bacteria | Proteobacteria | *Oligotropha* | 0.0017 | 0.0018 | 2.237 | 0.074 |
| Bacteria | Actinobacteria | *Olsenella* | 3.7410 | 3.6768 | 2.410 | 0.058 |
| Bacteria | Bacteroidetes | *Ornithobacterium* | 0.0032 | 0.0034 | 2.980 | 0.025 |
| Bacteria | Firmicutes | *Paenibacillus* | 0.1844 | 0.1841 | 4.142 | 0.005 |
| Bacteria | Proteobacteria | *Pantoea* | 0.0418 | 0.0446 | 7.050 | 0.000 |
| Fungi | Ascomycota | *Paracoccidioides* | 0.0012 | 0.0008 | 3.503 | 0.012 |
| Bacteria | Proteobacteria | *Paracoccus* | 0.0340 | 0.0502 | 2.391 | 0.060 |
| Bacteria | Firmicutes | *Parageobacillus* | 0.0092 | 0.0092 | 5.899 | 0.000 |
| Fungi | Ascomycota | *Paraphaeosphaeria* | 0.0308 | 0.0532 | 2.260 | 0.072 |
| Bacteria | Proteobacteria | *Pararhodospirillum* | 0.0032 | 0.0029 | 7.915 | 0.000 |
| Bacteria | Actinobacteria | *Parascardovia* | 0.0105 | 0.0099 | 2.512 | 0.050 |
| Fungi | Ascomycota | *Parastagonospora* | 0.0015 | 0.0019 | 3.115 | 0.021 |
| Bacteria | Proteobacteria | *Parvibaculum* | 0.0036 | 0.0035 | 7.991 | 0.000 |
| Bacteria | Proteobacteria | *Parvularcula* | 0.0014 | 0.0012 | 3.291 | 0.016 |
| Bacteria | Proteobacteria | *Pectobacterium* | 0.0091 | 0.0092 | 13.776 | 0.000 |
| Bacteria | Proteobacteria | *Pelobacter* | 0.0279 | 0.0284 | 7.730 | 0.000 |
| Bacteria | Chlorobi | *Pelodictyon* | 0.0107 | 0.0116 | 8.751 | 0.000 |
| Bacteria | Firmicutes | *Peptoniphilus* | 0.0036 | 0.0040 | 3.374 | 0.014 |
| Bacteria | Firmicutes | *Peptostreptococcus* | 0.0171 | 0.0178 | 9.187 | 0.000 |
| Other | Perkinsea | *Perkinsus* | 0.0094 | 0.0138 | 3.497 | 0.012 |
| Fungi | Basidiomycota | *Phanerochaete* | 0.0009 | 0.0015 | 5.313 | 0.001 |
| Fungi | Ascomycota | *Phialocephala* | 0.0038 | 0.0043 | 4.984 | 0.001 |
| Bacteria | Planctomycetes | *Phycisphaera* | 0.0108 | 0.0106 | 4.773 | 0.002 |
| Bacteria | Plantomycetes | *Pirellula* | 0.0022 | 0.0023 | 4.973 | 0.001 |
| Bacteria | Proteobacteria | *Planktomarina* | 0.0010 | 0.0010 | 5.162 | 0.001 |
| Bacteria | Firmicutes | *Planococcus* | 0.0114 | 0.0122 | 5.712 | 0.001 |
| Bacteria | Proteobacteria | *Plesiomonas* | 0.0023 | 0.0026 | 3.189 | 0.019 |
| Fungi | Ascomycota | *Pneumocystis* | 0.0139 | 0.0210 | 2.751 | 0.035 |
| Bacteria | Proteobacteria | *Polaromonas* | 0.0086 | 0.0091 | 6.738 | 0.000 |
| Bacteria | Bacteroidetes | *Pontibacter* | 0.0234 | 0.0260 | 2.480 | 0.052 |
| Bacteria | Proteobacteria | *Pragia* | 0.0018 | 0.0024 | 5.669 | 0.001 |
| Bacteria | Bacteroidetes | *Prevotella* | 43.9074 | 38.7977 | 2.686 | 0.039 |
| Bacteria | Actinobacteria | *Propionimicrobium* | 0.0010 | 0.0013 | 4.977 | 0.001 |
| Bacteria | Firmicutes | *Proteiniclasticum* | 0.0105 | 0.0101 | 4.988 | 0.001 |
| Bacteria | Proteobacteria | *Pseudoalteromonas* | 0.0144 | 0.0150 | 5.937 | 0.000 |
| Bacteria | Firmicutes | *Pseudobutyrivibrio* | 0.3465 | 0.5043 | 2.428 | 0.056 |
| Bacteria | Actinobacteria | *Pseudonocardia* | 0.0181 | 0.0180 | 3.358 | 0.015 |
| Bacteria | Thermotogae | *Pseudothermotoga* | 0.0018 | 0.0018 | 7.284 | 0.000 |
| Bacteria | Proteobacteria | *Pseudoxanthomonas* | 0.0223 | 0.0242 | 2.840 | 0.031 |
| Fungi | Basidiomycota | *Pseudozyma* | 0.0012 | 0.0010 | 3.355 | 0.015 |
| Bacteria | Bacteroidetes | *Psychroflexus* | 0.0021 | 0.0018 | 2.303 | 0.068 |
| Bacteria | Proteobacteria | *Pusillimonas* | 0.0042 | 0.0045 | 4.293 | 0.004 |
| Fungi | Ascomycota | *Pyrenophora* | 0.0049 | 0.0047 | 7.978 | 0.000 |
| Archaea | Euryarchaeota | *Pyrococcus* | 0.0016 | 0.0019 | 2.198 | 0.079 |
| Bacteria | Proteobacteria | *Rahnella* | 0.0049 | 0.0045 | 3.484 | 0.012 |
| Bacteria | Proteobacteria | *Raoultella* | 0.0115 | 0.0117 | 7.581 | 0.000 |
| Fungi | Ascomycota | *Rasamsonia* | 0.0038 | 0.0051 | 2.538 | 0.048 |
| Bacteria | Actinobacteria | *Renibacterium* | 0.0009 | 0.0012 | 2.474 | 0.053 |
| Bacteria | Proteobacteria | *Rhizobium* | 0.0907 | 0.0908 | 3.586 | 0.010 |
| Bacteria | Proteobacteria | *Rhodoluna* | 0.0012 | 0.0013 | 2.617 | 0.043 |
| Bacteria | Planctomycetes | *Rhodopirellula* | 0.0015 | 0.0016 | 3.660 | 0.009 |
| Bacteria | Proteobacteria | *Rhodopseudomonas* | 0.0216 | 0.0217 | 3.008 | 0.024 |
| Bacteria | Bacteroidetes | *Rhodothermus* | 0.0145 | 0.0148 | 4.946 | 0.002 |
| Fungi | Basidiomycota | *Rhodotorula* | 0.0029 | 0.0029 | 2.191 | 0.080 |
| Bacteria | Bacteroidetes | *Riemerella* | 0.0033 | 0.0032 | 6.440 | 0.000 |
| Bacteria | Cyanobacteria | *Rivularia* | 0.0270 | 0.0441 | 2.441 | 0.055 |
| Bacteria | Bacteroidetes | *Robiginitalea* | 0.0090 | 0.0102 | 2.347 | 0.063 |
| Bacteria | Proteobacteria | *Roseibacterium* | 0.0024 | 0.0027 | 7.471 | 0.000 |
| Bacteria | Planctomycetes | *Rubinisphaera* | 0.0024 | 0.0024 | 2.477 | 0.052 |
| Bacteria | Proteobacteria | *Rubrivivax* | 0.0110 | 0.0109 | 2.052 | 0.097 |
| Bacteria | Actinobacteria | *Rubrobacter* | 0.0103 | 0.0097 | 9.739 | 0.000 |
| Bacteria | Bacteroidetes | *Rufibacter* | 0.0274 | 0.0296 | 8.248 | 0.000 |
| Bacteria | Proteobacteria | *Ruminobacter* | 0.7182 | 0.7694 | 2.510 | 0.050 |
| Bacteria | Firmicutes | *Rummeliibacillus* | 0.0016 | 0.0011 | 4.559 | 0.003 |
| Bacteria | Actinobacteria | *Saccharomonospora* | 0.0174 | 0.0173 | 4.544 | 0.003 |
| Bacteria | Proteobacteria | *Saccharophagus* | 0.0013 | 0.0014 | 3.632 | 0.010 |
| Bacteria | Firmicutes | *Salimicrobium* | 0.0025 | 0.0024 | 3.929 | 0.006 |
| Bacteria | Bacteroidetes | *Salinibacter* | 0.0092 | 0.0090 | 3.676 | 0.009 |
| Bacteria | Firmicutes | *Salinicoccus* | 0.0034 | 0.0039 | 3.490 | 0.012 |
| Bacteria | Proteobacteria | *Salmonella* | 0.0209 | 0.0207 | 3.390 | 0.014 |
| Bacteria | Actinobacteria | *Sanguibacter* | 0.0102 | 0.0102 | 8.213 | 0.000 |
| Bacteria | Firmicutes | *Sarcina* | 0.5438 | 0.6335 | 3.276 | 0.016 |
| Bacteria | Actinobacteria | *Scardovia* | 0.0023 | 0.0032 | 2.308 | 0.067 |
| Fungi | Ascomycota | *Scedosporium* | 0.0147 | 0.0192 | 2.800 | 0.033 |
| Bacteria | Firmicutes | *Selenomonas* | 3.5309 | 3.7166 | 4.424 | 0.003 |
| Fungi | Ascomycota | *Setosphaeria* | 0.0035 | 0.0055 | 2.714 | 0.037 |
| Bacteria | Firmicutes | *Sharpea* | 1.6060 | 1.3330 | 3.462 | 0.013 |
| Bacteria | Proteobacteria | *Shewanella* | 0.0316 | 0.0338 | 3.823 | 0.007 |
| Bacteria | Proteobacteria | *Shigella* | 0.0010 | 0.0011 | 2.629 | 0.042 |
| Bacteria | Bacteroidetes | *Siansivirga* | 0.0026 | 0.0033 | 3.746 | 0.008 |
| Bacteria | Proteobacteria | *Sideroxydans* | 0.0121 | 0.0125 | 4.876 | 0.002 |
| Bacteria | Proteobacteria | *Simiduia* | 0.0026 | 0.0026 | 2.533 | 0.048 |
| Bacteria | Planctomycetes | *Singulisphaera* | 0.0032 | 0.0029 | 3.539 | 0.011 |
| Bacteria | Actinobacteria | *Slackia* | 0.1039 | 0.0937 | 7.195 | 0.000 |
| Bacteria | Fusobacteria | *Sneathia* | 0.0040 | 0.0045 | 3.367 | 0.014 |
| Bacteria | Spirochaetes | *Sphaerochaeta* | 0.0213 | 0.0238 | 8.419 | 0.000 |
| Bacteria | Bacteroidetes | *Sphingobacterium* | 0.0118 | 0.0118 | 2.909 | 0.028 |
| Bacteria | Proteobacteria | *Sphingobium* | 0.0303 | 0.0309 | 13.599 | 0.000 |
| Bacteria | Proteobacteria | *Sphingomonas* | 0.0491 | 0.0493 | 6.177 | 0.000 |
| Bacteria | Proteobacteria | *Sphingorhabdus* | 0.0025 | 0.0029 | 6.634 | 0.000 |
| Bacteria | Tenericutes | *Spiroplasma* | 0.0266 | 0.0306 | 4.675 | 0.002 |
| Bacteria | Bacteroidetes | *Spirosoma* | 0.0123 | 0.0143 | 12.249 | 0.000 |
| Fungi | Chytridiomycota | *Spizellomyces* | 0.0037 | 0.0054 | 2.933 | 0.027 |
| Bacteria | Actinobacteria | *Stackebrandtia* | 0.0047 | 0.0044 | 4.094 | 0.005 |
| Bacteria | Cyanobacteria | *Stanieria* | 0.0014 | 0.0011 | 2.141 | 0.085 |
| Bacteria | Firmicutes | *Staphylococcus* | 0.0453 | 0.0508 | 7.167 | 0.000 |
| Bacteria | Proteobacteria | *Starkeya* | 0.0047 | 0.0038 | 2.543 | 0.048 |
| Bacteria | Proteobacteria | *Stigmatella* | 0.0109 | 0.0105 | 5.732 | 0.001 |
| Bacteria | Actinobacteria | *Streptomyces* | 0.2180 | 0.2102 | 2.182 | 0.081 |
| Bacteria | Firmicutes | *Succiniclasticum* | 5.0539 | 5.4328 | 3.332 | 0.015 |
| Bacteria | Proteobacteria | *Succinivibrio* | 0.5665 | 0.9300 | 3.102 | 0.021 |
| Bacteria | Proteobacteria | *Sulfuricella* | 0.0037 | 0.0044 | 2.102 | 0.090 |
| Bacteria | Proteobacteria | *Sulfuritalea* | 0.0085 | 0.0094 | 3.565 | 0.011 |
| Bacteria | Firmicutes | *Syntrophomonas* | 0.0032 | 0.0031 | 2.588 | 0.045 |
| Bacteria | Firmicutes | *Syntrophothermus* | 0.0017 | 0.0018 | 2.238 | 0.074 |
| Bacteria | Proteobacteria | *Taylorella* | 0.0015 | 0.0012 | 3.089 | 0.022 |
| Bacteria | Firmicutes | *Tepidanaerobacter* | 0.0047 | 0.0048 | 2.974 | 0.025 |
| Bacteria | Proteobacteria | *Teredinibacter* | 0.0049 | 0.0053 | 3.489 | 0.012 |
| Bacteria | Firmicutes | *Terribacillus* | 0.0023 | 0.0018 | 2.113 | 0.089 |
| Bacteria | Acidobacteria | *Terriglobus* | 0.0023 | 0.0026 | 4.002 | 0.006 |
| Bacteria | Firmicutes | *Tetragenococcus* | 0.0092 | 0.0095 | 3.552 | 0.011 |
| Protist | Alveolata | *Tetrahymena* | 0.0020 | 0.0019 | 3.326 | 0.015 |
| Fungi | Ascomycota | *Tetrapisispora* | 0.0596 | 0.0986 | 2.164 | 0.083 |
| Bacteria | Proteobacteria | *Thalassolituus* | 0.0145 | 0.0218 | 2.313 | 0.067 |
| Bacteria | Proteobacteria | *Thalassospira* | 0.0046 | 0.0056 | 2.882 | 0.029 |
| Bacteria | Proteobacteria | *Thalassotalea* | 0.0028 | 0.0030 | 2.952 | 0.026 |
| Protist | Alveolata | *Theileria* | 0.0136 | 0.0213 | 2.598 | 0.044 |
| Bacteria | Firmicutes | *Thermacetogenium* | 0.0042 | 0.0049 | 2.123 | 0.088 |
| Bacteria | Firmicutes | *Thermaerobacter* | 0.0172 | 0.0170 | 8.567 | 0.000 |
| Bacteria | Firmicutes | *Thermoanaerobacter* | 0.0109 | 0.0151 | 3.300 | 0.016 |
| Bacteria | Thermobaculum | *Thermobaculum* | 0.0013 | 0.0015 | 4.595 | 0.002 |
| Bacteria | Firmicutes | *Thermodesulfobium* | 0.0018 | 0.0019 | 4.198 | 0.004 |
| Bacteria | Nitrospirae | *Thermodesulfovibrio* | 0.0022 | 0.0022 | 7.556 | 0.000 |
| Bacteria | Chloroflexi | *Thermomicrobium* | 0.0030 | 0.0026 | 2.944 | 0.027 |
| Bacteria | Actinobacteria | *Thermomonospora* | 0.0052 | 0.0047 | 2.404 | 0.058 |
| Bacteria | Firmicutes | *Thermosediminibacter* | 0.0025 | 0.0025 | 10.852 | 0.000 |
| Bacteria | Aquificae | *Thermosulfidibacter* | 0.0020 | 0.0024 | 2.175 | 0.081 |
| Bacteria | Cyanobacteria | *Thermosynechococcus* | 0.0030 | 0.0021 | 6.162 | 0.000 |
| Bacteria | Deinococcus-Thermus | *Thermus* | 0.0198 | 0.0202 | 3.668 | 0.009 |
| Bacteria | Proteobacteria | *Thioalkalivibrio* | 0.0451 | 0.0470 | 3.788 | 0.008 |
| Bacteria | Proteobacteria | *Thiocystis* | 0.0152 | 0.0157 | 3.793 | 0.008 |
| Bacteria | Proteobacteria | *Thioflavicoccus* | 0.0159 | 0.0163 | 2.105 | 0.090 |
| Bacteria | Proteobacteria | *Thiomonas* | 0.0100 | 0.0103 | 6.762 | 0.000 |
| Fungi | Basidiomycota | *Tilletiaria* | 0.0029 | 0.0031 | 4.718 | 0.002 |
| Bacteria | Spirochaetes | *Treponema* | 0.6346 | 0.7900 | 6.800 | 0.000 |
| Bacteria | Cyanobacteria | *Trichodesmium* | 0.0018 | 0.0032 | 3.031 | 0.023 |
| Bacteria | Cyanobacteria | *Trichormus* | 0.0080 | 0.0160 | 3.287 | 0.016 |
| Fungi | Basidiomycota | *Trichosporon* | 0.1180 | 0.0816 | 2.830 | 0.031 |
| Bacteria | Firmicutes | *Tyzzerella* | 0.0023 | 0.0025 | 2.117 | 0.089 |
| Fungi | Ascomycota | *Uncinocarpus* | 0.0118 | 0.0126 | 3.763 | 0.008 |
| Bacteria | Tenericutes | *Ureaplasma* | 0.0030 | 0.0029 | 3.347 | 0.015 |
| Fungi | Basidiomycota | *Ustilago* | 0.0033 | 0.0036 | 4.264 | 0.004 |
| Bacteria | Firmicutes | *Vagococcus* | 0.0023 | 0.0023 | 2.903 | 0.028 |
| Bacteria | Proteobacteria | *Variovorax* | 0.0272 | 0.0276 | 7.154 | 0.000 |
| Bacteria | Proteobacteria | *Verminephrobacter* | 0.0034 | 0.0038 | 2.807 | 0.032 |
| Bacteria | Verrucomicrobia | *Verrucomicrobium* | 0.0104 | 0.0107 | 2.102 | 0.090 |
| Bacteria | Proteobacteria | *Vibrio* | 0.0456 | 0.0508 | 2.627 | 0.042 |
| Bacteria | Firmicutes | *Virgibacillus* | 0.0044 | 0.0048 | 4.334 | 0.004 |
| Fungi | Basidiomycota | *Wallemia* | 0.0037 | 0.0056 | 3.208 | 0.018 |
| Bacteria | Bacteroidetes | *Weeksella* | 0.0015 | 0.0014 | 4.352 | 0.003 |
| Bacteria | Firmicutes | *Weissella* | 0.0030 | 0.0035 | 7.612 | 0.000 |
| Bacteria | Proteobacteria | *Wenzhouxiangella* | 0.0183 | 0.0193 | 3.604 | 0.010 |
| Bacteria | Proteobacteria | *Xanthobacter* | 0.0112 | 0.0111 | 3.491 | 0.012 |
| Bacteria | Proteobacteria | *Xanthomonas* | 0.2273 | 0.4064 | 3.172 | 0.019 |
| Fungi | Ascomycota | *Yamadazyma* | 0.0011 | 0.0019 | 3.030 | 0.023 |
| Bacteria | Proteobacteria | *Yangia* | 0.0039 | 0.0042 | 5.363 | 0.001 |
| Bacteria | Proteobacteria | *Zhongshania* | 0.0041 | 0.0036 | 8.169 | 0.000 |
| Bacteria | Proteobacteria | *Zymomonas* | 0.0027 | 0.0029 | 4.252 | 0.004 |

Table S3: PLS results (All VIP values) evaluating the impact of the volatile fatty acids, the acetate-to-propionate ratio and the genes to explain variation in feed efficiency (FCR and RFI).

| **Variable** | **FCR without genes** | | **FCR with genes** | | **RFI without genes** | | **RFI with genes** | |
| --- | --- | --- | --- | --- | --- | --- | --- | --- |
| **Genes/VFA/ratio** | **VIP** | **Coef.** | **VIP** | **Coef.** | **VIP** | **Coef.** | **VIP** | **Coef.** |
| Acetate | **0.87** | **-0.11** | 0.69 | -0.04 | 0.43 | -0.01 | 0.47 | 0.47 |
| Propionate | 0.69 | 0.06 | 0.40 | 0.02 | 0.66 | -0.05 | 0.53 | 0.53 |
| Iso-butyrate | 0.50 | -0.03 | 0.79 | -0.01 | 0.51 | -0.03 | 0.29 | 0.29 |
| butyrate | 0.77 | 0.03 | 0.73 | 0.01 | **0.84** | **0.10** | 0.68 | 0.68 |
| Iso-valerate | 0.52 | -0.04 | 0.34 | -0.02 | 0.76 | 0.07 | 0.54 | 0.54 |
| Valerate | 0.25 | 0.02 | 0.53 | 0.01 | 0.34 | -0.01 | 0.41 | 0.41 |
| Branched Chain | 0.75 | -0.03 | 0.34 | -0.01 | **1.02** | **0.12** | **0.90** | **0.90** |
| A:P ratio | 0.62 | -0.05 | 0.33 | -0.02 | 0.52 | 0.03 | 0.38 | 0.38 |
| K01206 | N.D. | N.D. | 0.32 | 0.02 | N.D. | N.D. | 0.22 | 0.22 |
| K01654 | N.D. | N.D. | 0.79 | -0.05 | N.D. | N.D. | **1.13** | **1.13** |
| K01818 | N.D. | N.D. | 0.62 | -0.03 | N.D. | N.D. | 0.49 | 0.49 |
| K01835 | N.D. | N.D. | 0.74 | -0.05 | N.D. | N.D. | **0.92** | **0.92** |
| K01840 | N.D. | N.D. | **1.10** | **-0.06** | N.D. | N.D. | **0.90** | **0.90** |
| K01993 | N.D. | N.D. | **1.02** | **0.03** | N.D. | N.D. | **1.00** | **1.00** |
| K02005 | N.D. | N.D. | 0.78 | -0.04 | N.D. | N.D. | 0.68 | 0.68 |
| K02283 | N.D. | N.D. | 1.11 | 0.04 | N.D. | N.D. | **1.21** | **1.21** |
| K02377 | N.D. | N.D. | 0.68 | 0.03 | N.D. | N.D. | **0.97** | **0.97** |
| K02390 | N.D. | N.D. | 0.77 | 0.00 | N.D. | N.D. | **1.31** | **1.31** |
| K02392 | N.D. | N.D. | **1.10** | **-0.01** | N.D. | N.D. | **1.17** | **1.17** |
| K02396 | N.D. | N.D. | **1.16** | **-0.01** | N.D. | N.D. | **1.24** | **1.24** |
| K02400 | N.D. | N.D. | **0.95** | **-0.02** | N.D. | N.D. | **0.85** | **0.85** |
| K02406 | N.D. | N.D. | **0.97** | **0.01** | N.D. | N.D. | **1.22** | **1.22** |
| K02407 | N.D. | N.D. | **1.14** | **-0.03** | N.D. | N.D. | **0.89** | **0.89** |
| K02410 | N.D. | N.D. | 0.70 | -0.02 | N.D. | N.D. | **1.90** | **1.90** |
| K02412 | N.D. | N.D. | **0.89** | **0.05** | N.D. | N.D. | **0.91** | **0.91** |
| K02429 | N.D. | N.D. | 0.21 | -0.01 | N.D. | N.D. | **0.93** | **0.93** |
| K02454 | N.D. | N.D. | **1.72** | **0.11** | N.D. | N.D. | **1.35** | **1.35** |
| K02652 | N.D. | N.D. | **1.15** | **0.06** | N.D. | N.D. | **1.29** | **1.29** |
| K02653 | N.D. | N.D. | **1.50** | **0.09** | N.D. | N.D. | **1.45** | **1.45** |
| K02662 | N.D. | N.D. | **1.27** | **0.00** | N.D. | N.D. | 0.75 | 0.75 |
| K02666 | N.D. | N.D. | **1.16** | **0.01** | N.D. | N.D. | 0.77 | 0.77 |
| K03205 | N.D. | N.D. | **2.16** | **0.12** | N.D. | N.D. | 0.71 | 0.71 |
| K06442 | N.D. | N.D. | **1.03** | **0.05** | N.D. | N.D. | **1.16** | **1.16** |
| K11068 | N.D. | N.D. | 0.27 | 0.00 | N.D. | N.D. | 0.78 | 0.78 |
| K11907 | N.D. | N.D. | **0.90** | **-0.05** | N.D. | N.D. | 0.22 | 0.22 |

In bold are variables with a VIP>0.8. N.D.: Non-determined. Coef.: Coefficient of regression for PLS results. VFA: Volattile fatty acids; A:P ratio: Acetate-to-Propionate ratio.

Table S4: Identification of microbial genera correlated with selected genes explaining variation in feed efficiency (FCR and RFI) using PLS analysis (VIP>0.8).

| **Gene K01654** | **% of variability = 44%** | **K01654 correlated with RFI** |
| --- | --- | --- |
| **VIP** | **Coefficient** | **MAG-Genus** |
| 1.46757 | 0.13199 | *Renibacterium* |
| 1.4456 | 0.08032 | *Fibroporia* |
| 1.35024 | 0.12633 | *Teredinibacter* |
| 1.28514 | 0.10977 | *Trichosporon* |
| 1.12497 | 0.11543 | *Brevibacterium* |
| 1.11496 | 0.1037 | *Methyloversatilis* |
| 1.04955 | 0.0033 | *Idiomarina* |
| 1.01366 | -0.07886 | *Methanosphaera* |
| 1.00761 | 0.09533 | *Saccharopolyspora* |
| 0.99829 | 0.02366 | *Nitratiruptor* |
| 0.98731 | 0.09803 | *Orientia* |
| 0.98496 | 0.00539 | *Paraglaciecola* |
| 0.98269 | 0.07341 | *Oribacterium* |
| 0.97747 | 0.00157 | *Lawsonia* |
| 0.90205 | 0.05969 | *Candidatus Methylopumilus* |
| 0.90149 | 0.00589 | *Succinivibrio* |
| 0.89087 | 0.0192 | *Sulfuricella* |
| 0.87172 | -0.07868 | *Pusillimonas* |
| 0.85886 | 0.08002 | *Stackebrandtia* |
| 0.84405 | -0.08127 | *Desulfocapsa* |
| 0.84268 | -0.06557 | *Eubacterium* |
| 0.83952 | 0.04944 | *Acetoanaerobium* |
| 0.81704 | -0.03191 | *Treponema* |
| **Gene K01840** | **% of variability = 56%** | **K01840 correlated with FCR** |
| **VIP** | **Coefficient** | **MAG-Genus** |
| 1.28901 | 0.07876 | *Pragia* |
| 1.27683 | 0.11128 | *Pseudopedobacter* |
| 1.26218 | 0.11483 | *Gilliamella* |
| 1.25356 | -0.06924 | *Chromohalobacter* |
| 1.24941 | -0.04076 | *Parachlamydia* |
| 1.1812 | -0.06216 | *Odoribacter* |
| 1.15812 | 0.10073 | *Leptospira* |
| 1.08849 | -0.03487 | *Kandleria* |
| 1.07608 | -0.0574 | *Caldanaerobacter* |
| 1.0655 | -0.04113 | *Runella* |
| 1.0501 | 0.06474 | *Oscillatoria* |
| 1.03109 | 0.10808 | *Carboxydothermus* |
| 1.01001 | 0.10158 | *Emticicia* |
| 0.9847 | 0.10459 | *Glutamicibacter* |
| 0.98453 | 0.07129 | *Succiniclasticum* |
| 0.9624 | 0.0403 | *Geoglobus* |
| 0.92583 | 0.09916 | *Aequorivita* |
| 0.90337 | -0.00715 | *Stanieria* |
| 0.85463 | 0.03565 | *Cyclobacterium* |
| 0.8451 | -0.03864 | *Flavisolibacter* |
| 0.83294 | 0.06676 | *Pseudohongiella* |
| **Gene K02005** | **% of variability = 60%** | **K02005 correlated with FCR** |
| **VIP** | **Coefficient** | **MAG-Genus** |
| 1.47358 | -0.1076 | *Chromohalobacter* |
| 1.4707 | 0.09539 | *Pseudopedobacter* |
| 1.43549 | 0.08635 | *Ochrobactrum* |
| 1.33575 | 0.0802 | *Carboxydothermus* |
| 1.1931 | 0.06408 | *Stenotrophomonas* |
| 1.16274 | 0.04689 | *Verrucosispora* |
| 1.15485 | -0.07826 | *Parachlamydia* |
| 1.15124 | 0.06411 | *Mesoplasma* |
| 1.13032 | -0.08308 | *Flexistipes* |
| 1.12267 | -0.08243 | *Draconibacterium* |
| 1.0783 | -0.07858 | *Runella* |
| 1.04939 | 0.05273 | *Planktomarina* |
| 1.02466 | 0.06524 | *Cyclobacterium* |
| 1.02265 | 0.02776 | *Flavonifractor* |
| 1.02011 | 0.05782 | *Owenweeksia* |
| 1.00274 | 0.03263 | *Intestinimonas* |
| 0.99351 | 0.02794 | *Paracoccus* |
| 0.96909 | 0.04821 | *Sulfurimonas* |
| 0.95044 | -0.06986 | *Polynucleobacter* |
| 0.94441 | 0.02171 | *Fervidobacterium* |
| 0.94055 | 0.01343 | *Brucella* |
| 0.93243 | 0.0322 | *Riemerella* |
| 0.92695 | -0.06622 | *Fluviicola* |
| 0.91254 | 0.02023 | *Acholeplasma* |
| 0.90054 | 0.05069 | *Methanobrevibacter* |
| 0.89761 | 0.02547 | *Marinilactibacillus* |
| 0.89401 | -0.06287 | *Flavisolibacter* |
| 0.87677 | 0.00035 | *Brachybacterium* |
| 0.87325 | -0.06316 | *Beijerinckia* |
| 0.86867 | 0.05289 | *Gramella* |
| 0.86852 | 0.03748 | *Glutamicibacter* |
| 0.85194 | 0.03019 | *Gloeomargarita* |
| 0.84632 | -0.06167 | *Barnesiella* |
| 0.84184 | 0.01316 | *Sphingobacterium* |
| 0.83766 | 0.06157 | *Debaryomyces* |
| 0.83449 | 0.01669 | *Caulobacter* |
| 0.81532 | 0.0139 | *Shinella* |
| 0.80529 | -0.00287 | *Acidovorax* |
| **Gene K02383** | **% of variability = 63%** | **K02383 correlated with RFI** |
| **VIP** | **Coefficient** | **MAG-Genus** |
| 1.3548 | 0.10553 | *Phanerochaete* |
| 1.26718 | 0.09838 | *Parachlamydia* |
| 1.25682 | 0.08488 | *Eubacterium* |
| 1.24211 | 0.08578 | *Sphaerochaeta* |
| 1.21446 | 0.08784 | *Candidatus Methanomethylophilus* |
| 1.15596 | -0.09232 | *Succiniclasticum* |
| 1.15029 | -0.0934 | *Oscillatoria* |
| 1.14088 | 0.08885 | *Methanolacinia* |
| 1.10719 | 0.03986 | *Thiomonas* |
| 1.07212 | -0.08587 | *Candidatus Carsonella* |
| 1.06808 | 0.02907 | *Agrobacterium* |
| 1.06801 | -0.01844 | *Methanoregula* |
| 1.06155 | 0.0862 | *Xylella* |
| 1.04909 | -0.07165 | *Methyloversatilis* |
| 1.04345 | 0.02012 | *Arthrobacter* |
| 1.03744 | 0.01892 | *Defluviimonas* |
| 1.036 | -0.07359 | *Aequorivita* |
| 1.03494 | 0.0136 | *Alcanivorax* |
| 1.03279 | 0.02506 | *Pannonibacter* |
| 1.02565 | 0.02512 | *Variovorax* |
| 1.02526 | -0.08275 | *Sporosarcina* |
| 1.01974 | 0.02292 | *Brevundimonas* |
| 1.0185 | 0.02376 | *Rhizobium* |
| 0.98938 | -0.01611 | *Gordonia* |
| 0.98093 | 0.0171 | *Sphingomonas* |
| 0.97576 | 0.01997 | *Chlorobium* |
| 0.97228 | -0.07935 | *Leptospira* |
| 0.9685 | 0.01637 | *Cupriavidus* |
| 0.96372 | -0.05922 | *Sulfurovum* |
| 0.95935 | 0.023 | *Alicyclobacillus* |
| 0.93517 | -0.06591 | *Mesoplasma* |
| 0.91131 | -0.04076 | *Paracoccus* |
| 0.91078 | -0.06877 | *Nonlabens* |
| 0.89655 | -0.04325 | *Kurthia* |
| 0.89476 | -0.05722 | *Alloactinosynnema* |
| 0.89042 | -0.04434 | *Hoeflea* |
| 0.88445 | -0.07096 | *Gloeomargarita* |
| 0.88108 | -0.03379 | *Dechloromonas* |
| 0.87985 | -0.06577 | *Brachybacterium* |
| 0.87484 | -0.0571 | *Pelosinus* |
| 0.84681 | -0.06184 | *Nitratifractor* |
| 0.81416 | -0.06152 | *Carboxydothermus* |
| **Gene K02396** | **% of variability = 68%** | **K02396 correlated with RFI** |
| **VIP** | **Coefficient** | **MAG-Genus** |
| 1.41242 | -0.10739 | *Dechloromonas* |
| 1.20075 | -0.1029 | *Pelosinus* |
| 1.20074 | -0.09969 | *Synechocystis* |
| 1.18331 | -0.04863 | *Methanoregula* |
| 1.14512 | -0.10076 | *Nonlabens* |
| 1.12889 | -0.05974 | *Hydrogenophaga* |
| 1.12809 | 0.0765 | *Eubacterium* |
| 1.12369 | 0.10353 | *Cellulophaga* |
| 1.11741 | 0.04201 | *Prosthecochloris* |
| 1.11616 | 0.0161 | *Rhodomicrobium* |
| 1.10331 | -0.04727 | *Altererythrobacter* |
| 1.08979 | 0.02021 | *Porphyrobacter* |
| 1.08735 | 0.10318 | *Teredinibacter* |
| 1.08273 | 0.08361 | *Neomicrococcus* |
| 1.08039 | 0.01685 | *Pantoea* |
| 1.07585 | 0.08792 | *Phanerochaete* |
| 1.05505 | 0.03958 | *Dialister* |
| 1.04068 | 0.08457 | *Lysobacter* |
| 1.04066 | 0.02708 | *Syntrophobotulus* |
| 1.03847 | 0.03777 | *Allisonella* |
| 1.03665 | 0.04288 | *Glarea* |
| 1.02134 | 0.03138 | *Azorhizobium* |
| 1.00905 | 0.05676 | *Desulfocapsa* |
| 1.00313 | 0.03414 | *Cellvibrio* |
| 0.99707 | 0.07197 | *Cryptobacterium* |
| 0.9481 | 0.08829 | *Mucilaginibacter* |
| 0.91363 | -0.08608 | *Sporosarcina* |
| 0.91109 | 0.08436 | *Providencia* |
| 0.87497 | 0.03716 | *Sphaerochaeta* |
| 0.84386 | 0.0763 | *Xylella* |
| 0.82795 | 0.05492 | *Ndongobacter* |
| 0.81107 | -0.07733 | *Cladophialophora* |
| 0.8064 | -0.06996 | *Succiniclasticum* |
| 0.80378 | 0.05991 | *Pelagibaca* |
| **Gene K02410** | **% of variability = 62%** | **K02410 correlated with RFI** |
| **VIP** | **Coefficient** | **MAG-Genus** |
| 1.51931 | 0.10066 | *Robiginitalea* |
| 1.31567 | -0.0817 | *Fuerstia* |
| 1.21287 | 0.0771 | *Sulfurihydrogenibium* |
| 1.21046 | -0.06495 | *Anaerolinea* |
| 1.20787 | -0.08025 | *Methanosphaera* |
| 1.17643 | 0.07001 | *Microcystis* |
| 1.17483 | -0.07208 | *Algoriphagus* |
| 1.16866 | -0.07283 | *Vagococcus* |
| 1.15798 | 0.0692 | *Mucinivorans* |
| 1.15191 | -0.07539 | *Mesoplasma* |
| 1.11073 | -0.06124 | *Syntrophomonas* |
| 1.09492 | 0.05477 | *Kibdelosporangium* |
| 1.08262 | -0.05327 | *Verrucomicrobium* |
| 1.07752 | -0.0678 | *Bdellovibrio* |
| 1.07726 | 0.05933 | *Brenneria* |
| 1.04947 | 0.07582 | *Negativicoccus* |
| 1.04095 | 0.07015 | *Advenella* |
| 1.04042 | -0.00384 | *Candidatus Solibacter* |
| 1.03456 | 0.04122 | *Pirellula* |
| 1.03426 | 0.0562 | *Draconibacterium* |
| 1.03216 | 0.00266 | *Hymenobacter* |
| 1.00969 | 0.04956 | *Seonamhaeicola* |
| 0.99088 | 0.00224 | *Alistipes* |
| 0.98838 | -0.06936 | *Desulfurobacterium* |
| 0.95946 | 0.05562 | *Tannerella* |
| 0.95867 | 0.04762 | *Natrinema* |
| 0.94467 | 0.03592 | *Natronomonas* |
| 0.93603 | 0.03321 | *Gluconacetobacter* |
| 0.92534 | 0.00546 | *Rhodothermus* |
| 0.92356 | 0.03557 | *Micrococcus* |
| 0.91568 | 0.04322 | *Parageobacillus* |
| 0.90224 | 0.06677 | *Providencia* |
| 0.90041 | 0.01635 | *Methylobacillus* |
| 0.89779 | 0.01099 | *Lentibacillus* |
| 0.89571 | 0.05783 | *Dokdonia* |
| 0.86868 | 0.02602 | *Dyadobacter* |
| 0.86708 | 0.00947 | *Barnesiella* |
| 0.8646 | 0.00228 | *Catenulispora* |
| 0.85791 | 0.06344 | *Teredinibacter* |
| 0.84506 | 0.05551 | *Belliella* |
| 0.81966 | 0.05735 | *Thermosulfidibacter* |
| 0.81085 | 0.01954 | *Brevibacillus* |
| **Gene K02429** | **% of variability = 56%** | **K02429 correlated with FCR** |
| **VIP** | **Coefficient** | **MAG-Genus** |
| 1.62663 | 0.10806 | *Ochrobactrum* |
| 1.60934 | 0.10682 | *Stenotrophomonas* |
| 1.2327 | 0.0736 | *Sphingobacterium* |
| 1.20464 | 0.07014 | *Brucella* |
| 1.19534 | 0.07377 | *Syntrophothermus* |
| 1.16653 | -0.05089 | *Gemmatimonas* |
| 1.15856 | 0.06992 | *Caulobacter* |
| 1.13573 | 0.07276 | *Methanobrevibacter* |
| 1.08596 | 0.07109 | *Sarcina* |
| 1.07495 | 0.06613 | *Achromobacter* |
| 1.06988 | -0.03691 | *Rhodothermus* |
| 1.0572 | -0.06567 | *Flexistipes* |
| 1.04903 | -0.03795 | *Novosphingobium* |
| 1.04429 | -0.06861 | *Algibacter* |
| 1.04178 | -0.02635 | *Yangia* |
| 1.03435 | -0.06867 | *Methanohalophilus* |
| 1.03366 | 0.04991 | *Verrucosispora* |
| 1.02992 | 0.05751 | *Hahella* |
| 1.02536 | 0.04982 | *Candidatus Symbiobacter* |
| 1.02182 | 0.06815 | *Cyclobacterium* |
| 1.01652 | -0.04116 | *Alistipes* |
| 1.01168 | -0.04559 | *Aerococcus* |
| 1.00573 | -0.02888 | *Nakamurella* |
| 0.99877 | -0.02812 | *Arsenicicoccus* |
| 0.9935 | 0.03226 | *Nocardiopsis* |
| 0.99277 | -0.02993 | *Thermomonospora* |
| 0.98584 | -0.04427 | *Tistrella* |
| 0.97762 | 0.01614 | *Kocuria* |
| 0.97102 | 0.06308 | *Pseudopedobacter* |
| 0.96724 | -0.03286 | *Lentibacillus* |
| 0.96654 | 0.01215 | *Desulfarculus* |
| 0.95676 | -0.00931 | *Sphaerobacter* |
| 0.95278 | -0.03001 | *Desulfohalobium* |
| 0.94763 | -0.05816 | *Ndongobacter* |
| 0.94448 | 0.01073 | *Thermus* |
| 0.94431 | -0.03903 | *Salinibacter* |
| 0.92864 | -0.01615 | *Desulfurivibrio* |
| 0.9231 | 0.02118 | *Roseomonas* |
| 0.92123 | 0.00992 | *Meiothermus* |
| 0.9063 | 0.04119 | *Saccharothrix* |
| 0.90568 | -0.03221 | *Acidobacterium* |
| 0.89963 | 0.04297 | *Oligotropha* |
| 0.89535 | -0.02175 | *Planococcus* |
| 0.89489 | 0.01979 | *Limnochorda* |
| 0.8943 | 0.03798 | *Chloracidobacterium* |
| 0.89162 | 0.04412 | *Parvularcula* |
| 0.88875 | 0.01274 | *Methylobacillus* |
| 0.88685 | 0.01423 | *Deinococcus* |
| 0.88332 | -0.03679 | *Alloactinosynnema* |
| 0.88329 | 0.01021 | *Nocardia* |
| 0.86843 | 0.02601 | *Candidatu Solibacter* |
| 0.8617 | 0.03554 | *Planctomyces* |
| **Gene K02454** | **% of variability = 55%** | **K02454 correlated with FCR and RFI** |
| **VIP** | **Coefficient** | **MAG-Genus** |
| 1.64106 | 0.030043 | *Parachlamydia* |
| 1.47582 | 0.026885 | *Methanolacinia* |
| 1.44605 | 0.023782 | *Acidaminococcus* |
| 1.3673 | 0.024162 | *Hoyosella* |
| 1.34246 | 0.022318 | *Kytococcus* |
| 1.33039 | 0.022071 | *Arcanobacterium* |
| 1.31839 | 0.02354 | *Jeotgalibacillus* |
| 1.2751 | -0.02385 | *Sulfuricella* |
| 1.25476 | 0.02346 | *Ruminococcus* |
| 1.25101 | -0.01988 | *Synechocystis* |
| 1.24457 | 0.018065 | *Sphaerochaeta* |
| 1.20949 | 0.022297 | *Stanieria* |
| 1.20294 | 0.0213 | *Phanerochaete* |
| 1.19613 | 0.019874 | *Salinicoccus* |
| 1.17351 | 0.019357 | *Flammeovirga* |
| 1.17037 | -0.02089 | *Pragia* |
| 1.16662 | -0.01698 | *Lentzea* |
| 1.15238 | -0.02175 | *Cyanothece* |
| 1.14302 | 0.017601 | *Rhodovulum* |
| 1.13262 | -0.01799 | *Methyloversatilis* |
| 1.12924 | 0.019062 | *Beutenbergia* |
| 1.12823 | 0.0151 | *Libanicoccus* |
| 1.12268 | -0.02115 | *Candidatus Amoebophilus* |
| 1.12217 | 0.017801 | *Candidatus Methanomethylophilus* |
| 1.11631 | 0.013853 | *Acetobacterium* |
| 1.1003 | 0.018232 | *Kandleria* |
| 1.09542 | 0.020065 | *Pyrococcus* |
| 1.09144 | 0.015987 | *Chromohalobacter* |
| 1.08916 | -0.02038 | *Arthrospira* |
| 1.08522 | 0.018943 | *Magnetospira* |
| 1.08316 | 0.020283 | *Polynucleobacter* |
| 1.07968 | -0.0118 | *Paracoccus* |
| 1.07917 | 0.012522 | *Thalassospira* |
| 1.0752 | 0.011383 | *Mitsuokella* |
| 1.07424 | 0.015289 | *Caldanaerobacter* |
| 1.07302 | 0.016367 | *Fluviicola* |
| 1.06874 | 0.017674 | *Dehalococcoides* |
| 1.06414 | 0.017699 | *Runella* |
| 1.05889 | -0.01997 | *Plesiomonas* |
| 1.04674 | 0.017791 | *Ilumatobacter* |
| 1.0462 | -0.0091 | *Gordonia* |
| 1.04282 | 0.008249 | *Thiomonas* |
| 1.04157 | 0.008101 | *Coriobacterium* |
| 1.03624 | 0.009466 | *Desulfitobacterium* |
| 1.03412 | 0.012991 | *Thermodesulfobium* |
| 1.03329 | -0.00589 | *Martelella* |
| 1.03013 | 0.012272 | *Atopobium* |
| 1.02927 | -0.01802 | *Peptoclostridium* |
| 1.02235 | 0.014693 | *Formosa* |
| 1.02204 | -0.01198 | *Kurthia* |
| 1.02202 | 0.00404 | *Paenibacillus* |
| 1.0194 | 0.000824 | *Arthrobacter* |
| 1.01937 | 0.007455 | *Thiobacimonas* |
| 1.01908 | -0.01476 | *Ammonifex* |
| 1.0182 | 0.018967 | *Parastagonospora* |
| 1.01789 | -0.00937 | *Rhodanobacter* |
| 1.01666 | 0.008366 | *Desulfosporosinus* |
| 1.01635 | 0.006554 | *Frankia* |
| 1.01552 | -0.01911 | *Oscillatoria* |
| 1.00313 | -0.00455 | *Aminomonas* |
| 1.00043 | 0.009487 | *Allisonella* |
| 0.99775 | -7.3E-05 | *Corynebacterium* |
| 0.99701 | 0.014469 | *Spiribacter* |
| 0.99515 | 0.011968 | *Megamonas* |
| 0.99431 | 0.012669 | *Zhongshania* |
| 0.99141 | 0.00751 | *Comamonas* |
| 0.98596 | 0.000154 | *Sphingomonas* |
| 0.98189 | -0.01675 | *Nitratifractor* |
| 0.9804 | -0.01821 | *Fibroporia* |
| 0.97942 | 0.007032 | *Peptostreptococcus* |
| 0.97869 | 0.007161 | *Sphingobium* |
| 0.97566 | -0.01676 | *Cryobacterium* |
| 0.97494 | 0.013312 | *Bacillus* |
| 0.97493 | 0.011019 | *Eubacterium* |
| 0.97399 | -0.00396 | *Stigmatella* |
| 0.97357 | -0.0064 | *Thioalkalivibrio* |
| 0.97278 | -0.00053 | *Brevundimonas* |
| 0.97176 | 0.009703 | *Lactococcus* |
| 0.97136 | -0.00325 | *Alicyclobacillus* |
| 0.9705 | 0.014441 | *Melissococcus* |
| 0.96856 | 0.00318 | *Desulfococcus* |
| 0.96853 | 0.015842 | *Halobacillus* |
| 0.96847 | 0.001055 | *Archangium* |
| 0.96741 | -0.01735 | *Thalassolituus* |
| 0.96617 | 0.000037 | *Agrobacterium* |
| 0.96502 | 0.018036 | *Pseudanabaena* |
| 0.9633 | -0.00884 | *Heliobacterium* |
| 0.96301 | 0.013038 | *Saccharomonospora* |
| 0.95966 | 0.005109 | *Sanguibacter* |
| 0.95928 | -0.01512 | *Nonlabens* |
| 0.95878 | 0.011992 | *Sulfitobacter* |
| 0.95693 | -0.01278 | *Pusillimonas* |
| 0.95673 | 0.012481 | *Ndongobacter* |
| 0.95654 | 0.000113 | *Chlorobium* |
| 0.95587 | -0.00458 | *Methanoregula* |
| 0.95546 | 0.016022 | *Cyphellophora* |
| 0.95274 | 0.000139 | *Rhizobium* |
| 0.95148 | -0.00302 | *Cupriavidus* |
| 0.95029 | 0.017644 | *Flavisolibacter* |
| 0.94667 | -0.00133 | *Defluviimonas* |
| 0.94529 | 0.01535 | *Confluentimicrobium* |
| 0.94399 | -0.01564 | *Thermanaerovibrio* |
| 0.9418 | 0.000164 | *Variovorax* |
| 0.94127 | -0.01762 | *Gilliamella* |
| 0.9385 | 0.00038 | *Pannonibacter* |
| 0.93681 | -0.01566 | *Gregarina* |
| 0.93637 | -0.00686 | *Streptomyces* |
| 0.93243 | 0.01239 | *Anoxybacillus* |
| 0.92923 | -0.00796 | *Methanocella* |
| 0.9273 | 0.012207 | *Sphingorhabdus* |
| 0.92606 | -0.01132 | *Candidatus Phaeomarinobacter* |
| 0.92447 | -0.01718 | *Cryptococcus* |
| 0.92294 | -0.01144 | *Gluconacetobacter* |
| 0.92171 | 0.015324 | *Terrisporobacter* |
| 0.92114 | -0.00347 | *Thermaerobacter* |
| 0.91997 | -0.00087 | *Alcanivorax* |
| 0.9189 | -0.00206 | *Clavibacter* |
| 0.91831 | -0.00152 | *Castellaniella* |
| 0.91823 | 0.015366 | *Scardovia* |
| 0.91555 | -0.00921 | *Deinococcus* |
| 0.91528 | 0.012144 | *Caldisericum* |
| 0.91428 | -0.01249 | *Alloactinosynnema* |
| 0.91419 | -0.00945 | *Rhodoluna* |
| 0.91195 | -0.01668 | *Xenorhabdus* |
| 0.91119 | -0.00487 | *Cyanobium* |
| 0.91098 | 0.017174 | *Metschnikowia* |
| 0.9109 | -0.00646 | *Mycobacterium* |
| 0.90894 | -0.00475 | *Sinomonas* |
| 0.90755 | -0.016 | *Idiomarina* |
| 0.90644 | -0.00858 | *Pseudogulbenkiania* |
| 0.9063 | -0.0093 | *Bosea* |
| 0.90482 | -0.00369 | *Geobacter* |
| 0.90192 | -0.00829 | *Steroidobacter* |
| 0.90137 | -0.01143 | *Micromonospora* |
| 0.89846 | -0.00954 | *Polaromonas* |
| 0.89681 | -0.01036 | *Rhodoferax* |
| 0.89391 | 0.013195 | *Kozakia* |
| 0.89318 | -0.01567 | *Geoglobus* |
| 0.89102 | -0.00359 | *Halomonas* |
| 0.88878 | 0.015517 | *Mahella* |
| 0.88754 | -0.01281 | *Ketogulonicigenium* |
| 0.88636 | -0.00326 | *Azospira* |
| 0.8863 | 0.0146 | *Ehrlichia* |
| 0.88452 | -0.01664 | *Leptospira* |
| 0.88233 | -0.00435 | *Bradyrhizobium* |
| 0.88167 | -0.00494 | *Hydrogenophaga* |
| 0.87996 | -0.01569 | *Succiniclasticum* |
| 0.87972 | -0.00248 | *Anaeromyxobacter* |
| 0.87833 | 0.016572 | *Methanomicrobium* |
| 0.87682 | -0.01648 | *Haliscomenobacter* |
| 0.87638 | -0.0026 | *Chelatococcus* |
| 0.86822 | -0.00392 | *Xanthobacter* |
| 0.86723 | -0.00536 | *Asticcacaulis* |
| 0.86619 | -0.00924 | *Sphaerobacter* |
| 0.86478 | -0.00815 | *Dickeya* |
| 0.86459 | -0.00426 | *Azoarcus* |
| 0.86289 | -0.01435 | *Brachybacterium* |
| 0.85851 | -0.01466 | *Polymorphum* |
| 0.85307 | -0.01584 | *Guillardia* |
| 0.85117 | 0.014842 | *Nitrosomonas* |
| 0.85083 | -0.0151 | *Neospora* |
| **Gene K02652** | **% of variability = 67%** | **K02652 correlated with FCR and RFI** |
| **VIP** | **Coefficient** | **MAG-Genus** |
| 1.50141 | -0.08133 | *Leifsonia* |
| 1.49831 | -0.0897 | *Microbacterium* |
| 1.46431 | -0.07359 | *Paracoccus* |
| 1.37614 | 0.098301 | *Treponema* |
| 1.33767 | -0.08943 | *Synechocystis* |
| 1.27746 | -0.09431 | *Fibroporia* |
| 1.25665 | -0.08789 | *Rahnella* |
| 1.2424 | 0.089575 | *Fibrobacter* |
| 1.13947 | 0.084509 | *Megasphaera* |
| 1.09205 | -0.02914 | *Rubrobacter* |
| 1.07801 | -0.07769 | *Teredinibacter* |
| 1.05081 | -0.01107 | *Catenulispora* |
| 1.02069 | 0.040788 | *Ottowia* |
| 1.01454 | -0.01421 | *Parvibaculum* |
| 1.01307 | -0.07374 | *Sulfuricella* |
| 0.98357 | -0.01726 | *Sphingobium* |
| 0.98138 | 0.068513 | *Elusimicrobium* |
| 0.98017 | -0.01275 | *Dermacoccus* |
| 0.97983 | -0.00419 | *Sphaerobacter* |
| 0.96398 | 0.06878 | *Caldimicrobium* |
| 0.94548 | 0.014549 | *Meiothermus* |
| 0.9433 | -0.00924 | *Agromyces* |
| 0.94259 | -0.00614 | *Anaerolinea* |
| 0.94018 | 0.037067 | *Geitlerinema* |
| 0.93007 | 0.000772 | *Methylomicrobium* |
| 0.91604 | 0.004926 | *Arsenicicoccus* |
| 0.91444 | -0.00545 | *Eggerthella* |
| 0.90732 | 0.019336 | *Polaromonas* |
| 0.89297 | 0.032113 | *Acidobacterium* |
| 0.87119 | 0.060036 | *Simiduia* |
| 0.8673 | 0.008811 | *Planococcus* |
| 0.86482 | 0.010243 | *Rhodothermus* |
| 0.84443 | 0.023171 | *Pararhodospirillum* |
| 0.84197 | 0.013241 | *Chondromyces* |
| 0.84104 | 0.047915 | *Eubacterium* |
| 0.84011 | -0.06034 | *Idiomarina* |
| 0.83135 | 0.061156 | *Capnocytophaga* |
| 0.82212 | 0.050287 | *Candidatus Azobacteroides* |
| 0.81074 | 0.048656 | *Mobiluncus* |
| 0.8089 | 0.046865 | *Thermosynechococcus* |
| 0.80572 | 0.023312 | *Syntrophomonas* |
| 0.80261 | -0.05651 | *Maribacter* |
| 0.80212 | 0.028281 | *Pirellula* |
| 0.80144 | 0.008214 | *Ilumatobacter* |
| **Gene K02653** | **% of variability = 55%** | **K02653 correlated with FCR and RFI** |
| **VIP** | **Coefficient** | **MAG-Genus** |
| 1.26928 | -0.05724 | *Synechocystis* |
| 1.23774 | 0.054693 | *Jeotgalibacillus* |
| 1.22655 | -0.04133 | *Paracoccus* |
| 1.18481 | -0.0502 | *Aequorivita* |
| 1.17997 | -0.05643 | *Peptoclostridium* |
| 1.17192 | -0.03317 | *Haloferax* |
| 1.15705 | 0.008782 | *Acetobacterium* |
| 1.15533 | -0.05796 | *Sulfuricella* |
| 1.15475 | 0.056193 | *Capnocytophaga* |
| 1.14266 | -0.04593 | *Lentzea* |
| 1.13295 | 0.032655 | *Ilumatobacter* |
| 1.12979 | -0.05174 | *Butyrivibrio* |
| 1.12462 | -0.03388 | *Faecalibaculum* |
| 1.1111 | -0.04297 | *Rhodoluna* |
| 1.08272 | -0.0543 | *Fibroporia* |
| 1.08264 | -0.04291 | *Candidatus Phaeomarinobacter* |
| 1.07952 | -0.03645 | *Syntrophus* |
| 1.06101 | -0.0368 | *Croceicoccus* |
| 1.06018 | -0.03975 | *Salimicrobium* |
| 1.05021 | -0.02823 | *Martelella* |
| 1.03773 | -0.00384 | *Cloacibacillus* |
| 1.03472 | 0.017024 | *Sphaerochaeta* |
| 1.02763 | 0.026143 | *Magnetospira* |
| 1.02331 | 0.03772 | *Parastagonospora* |
| 1.02242 | 0.023979 | *Listeria* |
| 1.02171 | 0.040098 | *Pseudanabaena* |
| 1.02169 | -0.01296 | *Corynebacterium* |
| 1.02054 | -0.03278 | *Methanocella* |
| 1.01652 | -0.05102 | *Oscillatoria* |
| 1.01582 | -0.04222 | *Desulfobacca* |
| 1.00091 | -0.04459 | *Pragia* |
| 0.99585 | 0.017948 | *Confluentimicrobium* |
| 0.99378 | 0.017328 | *Sphingorhabdus* |
| 0.99341 | 0.014741 | *Salinicoccus* |
| 0.98117 | -0.0165 | *Arthrobacter* |
| 0.95513 | 0.034795 | *Rickettsia* |
| 0.95401 | 0.044223 | *Selenomonas* |
| 0.95394 | 0.044328 | *Megasphaera* |
| 0.94696 | 0.002993 | *Sphingobium* |
| 0.94401 | 0.030153 | *Ehrlichia* |
| 0.94378 | 0.038518 | *Metschnikowia* |
| 0.94156 | -0.02339 | *Aminomonas* |
| 0.94089 | 0.02269 | *Amphibacillus* |
| 0.94026 | 0.035228 | *Solibacillus* |
| 0.93868 | 0.046966 | *Magnetococcus* |
| 0.93589 | 0.027066 | *Pyrococcus* |
| 0.93124 | -0.04656 | *Gilliamella* |
| 0.92935 | 0.028377 | *Acidaminococcus* |
| 0.92266 | 0.032329 | *Kytococcus* |
| 0.91702 | 0.030132 | *Vagococcus* |
| 0.91663 | -0.02301 | *Stigmatella* |
| 0.91366 | 0.019469 | *Eubacterium* |
| 0.90526 | 0.02659 | *Nitrosomonas* |
| 0.8976 | 0.040351 | *Rhodopirellula* |
| 0.88121 | 0.026213 | *Formosa* |
| 0.86793 | 0.02639 | *Candidatus Symbiobacter* |
| 0.86751 | 0.038938 | *Oligotropha* |
| 0.86688 | 0.030346 | *Mahella* |
| 0.86617 | 0.025252 | *Terrisporobacter* |
| 0.86574 | -0.03805 | *Idiomarina* |
| 0.86491 | 0.032537 | *Hoyosella* |
| 0.85864 | 0.038205 | *Parachlamydia* |
| 0.84971 | 0.029929 | *Polynucleobacter* |
| 0.8471 | -0.03413 | *Gregarina* |
| 0.84657 | 0.026929 | *Wenyingzhuangia* |
| 0.83816 | 0.032075 | *Runella* |
| **Gene K03205** | **% of variability = 67%** | **K03205 correlated with FCR** |
| **VIP** | **Coefficient** | **MAG-Genus** |
| 1.67297 | 0.18265 | *Elusimicrobium* |
| 1.55565 | 0.16888 | *Trichomonas* |
| 1.3254 | 0.13553 | *Methanosphaera* |
| 1.23489 | 0.12472 | *Fuerstia* |
| 1.1934 | 0.11312 | *Halogeometricum* |
| 1.1871 | 0.12495 | *Peptoclostridium* |
| 1.11836 | 0.0928 | *Oceanithermus* |
| 1.11234 | -0.07815 | *Verrucosispora* |
| 1.10872 | 0.09962 | *Desulfohalobium* |
| 1.03404 | 0.1055 | *Treponema* |
| 0.99757 | -0.07359 | *Punctularia* |
| 0.98027 | -0.05667 | *Candidatus Symbiobacter* |
| 0.97951 | -0.03684 | *Yangia* |
| 0.97629 | -0.03318 | *Verrucomicrobium* |
| 0.97068 | -0.03435 | *Paracoccus* |
| 0.96583 | -0.00706 | *Meiothermus* |
| 0.95935 | 0.00019 | *Thermus* |
| 0.95238 | -0.02484 | *Nocardia* |
| 0.95016 | -0.05169 | *Thermanaerovibrio* |
| 0.93875 | 0.00834 | *Nocardiopsis* |
| 0.93726 | -0.0761 | *Yarrowia* |
| 0.93508 | 0.10211 | *Beijerinckia* |
| 0.93142 | -0.00984 | *Intrasporangium* |
| 0.92513 | -0.0266 | *Micrococcus* |
| 0.9251 | 0.09634 | *Candidatus Cardinium* |
| 0.92114 | -0.03085 | *Corynebacterium* |
| 0.91917 | 0.00503 | *Sphaerobacter* |
| 0.91732 | -0.00353 | *Dermacoccus* |
| 0.91535 | -0.05308 | *Glutamicibacter* |
| 0.91114 | -0.00536 | *Alicycliphilus* |
| 0.9032 | -0.00448 | *Actinomyces* |
| 0.90286 | -0.03024 | *Haladaptatus* |
| 0.90274 | 0.00462 | *Catenulispora* |
| 0.895 | -0.07971 | *Thioalkalimicrobium* |
| 0.87241 | -0.01713 | *Frankia* |
| 0.80856 | 0.08845 | *Guillardia* |
| **Gene K06442** | **% of variability = 64%** | **K06442 correlated with RFI** |
| **VIP** | **Coefficient** | **MAG-Genus** |
| 1.40848 | -0.13429 | *Aequorivita* |
| 1.35501 | -0.12621 | *Pelosinus* |
| 1.34471 | -0.13421 | *Nonlabens* |
| 1.19522 | 0.10306 | *Phanerochaete* |
| 1.16082 | 0.04872 | *Sphaerochaeta* |
| 1.15908 | 0.06751 | *Eubacterium* |
| 1.14541 | 0.07942 | *Ndongobacter* |
| 1.14427 | 0.02999 | *Acidaminococcus* |
| 1.1321 | 0.03714 | *Allisonella* |
| 1.10592 | 0.03704 | *Dialister* |
| 1.09445 | 0.03483 | *Desulfitobacterium* |
| 1.07285 | 0.05355 | *Prosthecochloris* |
| 1.06808 | 0.11248 | *Streptococcus* |
| 1.0447 | 0.06722 | *Parachlamydia* |
| 1.03962 | -0.04753 | *Methanoregula* |
| 1.00627 | 0.03505 | *Rhodovulum* |
| 0.99955 | -0.08977 | *Succiniclasticum* |
| 0.98003 | 0.05448 | *Candidatus Methanomethylophilus* |
| 0.97986 | 0.02746 | *Flammeovirga* |
| 0.97651 | -0.10252 | *Brachybacterium* |
| 0.95923 | -0.10103 | *Sporosarcina* |
| 0.92875 | 0.0906 | *Mucilaginibacter* |
| 0.90282 | 0.04654 | *Methanolacinia* |
| 0.86289 | 0.04324 | *Hoyosella* |
| 0.82703 | -0.07836 | *Cyanothece* |
| 0.82018 | -0.08607 | *Verrucosispora* |
| 0.81098 | 0.06789 | *Filifactor* |
| 0.80892 | -0.01384 | *Thioalkalivibrio* |
| **Gene K11068** | **% of variability = 53%** | **K11068 correlated with FCR** |
| **VIP** | **Coefficient** | **MAG-Genus** |
| 1.45363 | 0.13575 | *Stenotrophomonas* |
| 1.4302 | 0.0784 | *Setosphaeria* |
| 1.3703 | -0.11208 | *Methanohalophilus* |
| 1.30457 | -0.09124 | *Aerococcus* |
| 1.29713 | 0.12112 | *Ochrobactrum* |
| 1.27863 | 0.1127 | *Bifidobacterium* |
| 1.14403 | -0.07934 | *Alloactinosynnema* |
| 1.12217 | -0.07727 | *Ketogulonicigenium* |
| 1.08531 | -0.08076 | *Halarchaeum* |
| 1.06038 | 0.09511 | *Wenyingzhuangia* |
| 1.05559 | 0.0946 | *Achromobacter* |
| 1.04256 | 0.09458 | *Caulobacter* |
| 1.04109 | 0.01223 | *Kazachstania* |
| 1.00196 | -0.07863 | *Algibacter* |
| 0.98567 | 0.01011 | *Methanocaldococcus* |
| 0.98465 | -0.00021 | *Ichthyophthirius* |
| 0.98398 | 0.00375 | *Dictyostelium* |
| 0.98157 | 0.00076 | *Dactylellina* |
| 0.9801 | 0.00035 | *Eutypa* |
| 0.97948 | -0.08066 | *Arachidicoccus* |
| 0.9774 | 0.0842 | *Lactobacillus* |
| 0.97326 | -0.00432 | *Pneumocystis* |
| 0.97136 | -0.00018 | *Spathaspora* |
| 0.97016 | -0.00282 | *Tetrahymena* |
| 0.96943 | -0.00147 | *Cryptosporidium* |
| 0.9688 | -0.00332 | *Lodderomyces* |
| 0.96723 | -0.00288 | *Naumovozyma* |
| 0.96657 | -0.00231 | *Plasmodium* |
| 0.96167 | -0.00366 | *Nosema* |
| 0.96039 | -0.00487 | *Blastomyces* |
| 0.95903 | -0.00397 | *Coccidioides* |
| 0.95798 | -0.00267 | *Kwoniella* |
| 0.95781 | -0.00423 | *Wickerhamomyces* |
| 0.95588 | -0.00651 | *Tetrapisispora* |
| 0.95453 | -0.00748 | *Entamoeba* |
| 0.95383 | -0.0049 | *Arthrobotrys* |
| 0.95195 | -0.00612 | *Paramecium* |
| 0.95072 | -0.00673 | *Candida* |
| 0.9476 | -0.00584 | *Trypanosoma* |
| 0.93881 | 0.0876 | *Sphingobacterium* |
| 0.93204 | -0.08506 | *Fibroporia* |
| 0.93155 | -0.00529 | *Perkinsus* |
| 0.92314 | -0.01326 | *Saccharomyces* |
| 0.91705 | -0.00472 | *Methanococcus* |
| 0.9163 | -0.00141 | *Marssonina* |
| 0.9162 | 0.08556 | *Actinotignum* |
| 0.8556 | -0.06762 | *Neofusicoccum* |
| 0.84677 | -0.07826 | *Serpula* |

Table S5: Identification of microbial genera explaining variation in feed efficiency (FCR and RFI) using PLS analysis (VIP>0.8).

| **Genera correlated with FCR** | **% of variability = 60%** |  |
| --- | --- | --- |
| **VIP** | **Coefficient** | **MAG-Genus** |
| 1.17707 | 0.054526 | *Salinicoccus* |
| 1.15347 | 0.066595 | *Algibacter* |
| 1.14811 | 0.066915 | *Elusimicrobium* |
| 1.13841 | 0.062978 | *Methanohalophilus* |
| 1.13641 | 0.070354 | *Petrimonas* |
| 1.12723 | 0.053731 | *Magnetospira* |
| 1.10992 | 0.036974 | *Sphaerochaeta* |
| 1.09365 | 0.032387 | *Acetobacterium* |
| 1.07659 | 0.061264 | *Treponema* |
| 1.06417 | 0.052418 | *Flammeovirga* |
| 1.06258 | 0.039686 | *Salinispora* |
| 1.05989 | 0.040211 | *Faecalitalea* |
| 1.04606 | 0.053165 | *Scardovia* |
| 1.03344 | 0.03624 | *Bdellovibrio* |
| 1.03303 | 0.045747 | *Acidaminococcus* |
| 1.03074 | -0.05251 | *Kiritimatiella* |
| 1.02754 | 0.051396 | *Methanosphaera* |
| 1.01422 | 0.048409 | *Desulfobacula* |
| 1.00892 | -0.05978 | *Methyloversatilis* |
| 1.0058 | -0.03313 | *Rubrobacter* |
| 1.00272 | -0.04245 | *Gordonibacter* |
| 1.00104 | 0.019084 | *Melioribacter* |
| 0.998 | 0.034111 | *Ilumatobacter* |
| 0.98954 | -0.01524 | *Frankia* |
| 0.98491 | -0.011 | *Paenibacillus* |
| 0.9802 | 0.061557 | *Natronolimnobius* |
| 0.9783 | 0.037683 | *Tyzzerella* |
| 0.97775 | -0.03058 | *Martelella* |
| 0.97399 | -0.0002 | *Coriobacterium* |
| 0.96362 | 0.015601 | *Pelobacter* |
| 0.96263 | -0.00492 | *Ethanoligenens* |
| 0.9584 | -0.03378 | *Stigmatella* |
| 0.95555 | -0.02511 | *Archangium* |
| 0.95356 | -0.05989 | *Sarcina* |
| 0.94979 | -0.03089 | *Stackebrandtia* |
| 0.94269 | -0.01213 | *Actinomyces* |
| 0.94167 | 0.051468 | *Ornithobacterium* |
| 0.93439 | -0.05519 | *Heterobasidion* |
| 0.93294 | -0.00713 | *Jeongeupia* |
| 0.93263 | -0.05116 | *Methanobrevibacter* |
| 0.90771 | -0.01742 | *Desulfomicrobium* |
| 0.90214 | -0.05634 | *Polymorphum* |
| 0.896 | 0.055824 | *Serpula* |
| 0.89472 | 0.05457 | *Trichomonas* |
| 0.86763 | -0.00285 | *Bifidobacterium* |
| 0.82178 | -0.05109 | *Phenylobacterium* |
| 0.82036 | -0.02071 | *Succiniclasticum* |
| 0.65412 | -0.00215 | *Lactobacillus* |
| **Genera correlated with RFI** | **% of variability = 60%** |  |
| **VIP** | **Coefficient** | **MAG-Genus** |
| 1.59386 | 0.051307 | *Synechocystis* |
| 1.41884 | 0.047396 | *Butyrivibrio* |
| 1.3582 | 0.039329 | *Hoeflea* |
| 1.35392 | 0.045423 | *Haloterrigena* |
| 1.31302 | 0.046967 | *Pseudobutyrivibrio* |
| 1.29119 | 0.045228 | *Sulfuricella* |
| 1.27182 | 0.039375 | *Leisingera* |
| 1.21752 | 0.036537 | *Thermacetogenium* |
| 1.21705 | 0.043829 | *Cladophialophora* |
| 1.20452 | 0.024264 | *Desulfococcus* |
| 1.19818 | 0.037445 | *Brachybacterium* |
| 1.1687 | 0.036215 | *Microcoleus* |
| 1.16662 | 0.041187 | *Fibroporia* |
| 1.15617 | 0.035653 | *Desulfotomaculum* |
| 1.14571 | 0.036522 | *Emiliania* |
| 1.13573 | 0.037379 | *Providencia* |
| 1.12065 | 0.035716 | *Robiginitalea* |
| 1.11948 | 0.036724 | *Collimonas* |
| 1.09432 | -0.03969 | *Thermosynechococcus* |
| 1.07193 | 0.038853 | *Paraglaciecola* |
| 1.05992 | 0.010408 | *Arthrobacter* |
| 1.04396 | 0.037842 | *Idiomarina* |
| 1.04384 | 0.010132 | *Brevundimonas* |
| 1.03492 | 0.011393 | *Rhodanobacter* |
| 1.01612 | 0.035762 | *Sebaldella* |
| 1.01279 | 0.008641 | *Herbaspirillum* |
| 1.01214 | 0.036714 | *Coxiella* |
| 1.00675 | 0.036238 | *Aminobacterium* |
| 0.98373 | 0.035618 | *Hammondia* |
| 0.97242 | 0.001181 | *Alcanivorax* |
| 0.96846 | 0.004214 | *Chelatococcus* |
| 0.95537 | 0.00071 | *Variovorax* |
| 0.95084 | -0.00031 | *Agrobacterium* |
| 0.94853 | -0.00026 | *Pannonibacter* |
| 0.94663 | 0.001156 | *Halomonas* |
| 0.94513 | 0.001339 | *Rhizobium* |
| 0.93469 | 0.033898 | *Sarcina* |
| 0.93442 | -0.0007 | *Thioalkalivibrio* |
| 0.93339 | -0.00051 | *Aromatoleum* |
| 0.93095 | -0.0013 | *Microbulbifer* |
| 0.92598 | 0.033117 | *Pragia* |
| 0.9248 | 0.000113 | *Thiocystis* |
| 0.92465 | 0.002928 | *Halotalea* |
| 0.92398 | -0.00176 | *Azospira* |
| 0.92102 | 0.033375 | *Aureobasidium* |
| 0.91996 | 0.000482 | *Thioflavicoccus* |
| 0.91833 | -0.00289 | *Methyloceanibacter* |
| 0.9174 | -0.00661 | *Defluviimonas* |
| 0.91739 | 0.00067 | *Azoarcus* |
| 0.91524 | -0.00241 | *Xanthobacter* |
| 0.91371 | -0.00786 | *Sideroxydans* |
| 0.91323 | -0.00569 | *Wenzhouxiangella* |
| 0.91315 | -0.00783 | *Modestobacter* |
| 0.91112 | -0.00648 | *Castellaniella* |
| 0.91057 | -0.0057 | *Pantoea* |
| 0.91022 | -0.01485 | *Methanomassiliicoccus* |
| 0.90929 | -0.00428 | *Tilletiaria* |
| 0.90912 | -0.00684 | *Raoultella* |
| 0.90904 | -0.01034 | *Truepera* |
| 0.90884 | 0.000722 | *Tsukamurella* |
| 0.90676 | -0.00171 | *Edwardsiella* |
| 0.90542 | -0.00366 | *Sinomonas* |
| 0.90407 | -0.00665 | *Halosimplex* |
| 0.90171 | -0.01665 | *Porphyrobacter* |
| 0.89475 | -0.01109 | *Leptothrix* |
| 0.89247 | -0.02989 | *Cryptobacterium* |
| 0.89115 | -0.00982 | *Chthonomonas* |
| 0.88927 | -0.01947 | *Kalmanozyma* |
| 0.88476 | -0.00179 | *Azorhizobium* |
| 0.88389 | 0.032053 | *Grosmannia* |
| 0.87907 | 0.030663 | *Gregarina* |
| 0.87086 | -0.00994 | *Grimontia* |
| 0.8704 | 0.031326 | *Rasamsonia* |
| 0.86731 | -0.00957 | *Coraliomargarita* |
| 0.8562 | 0.031029 | *Lawsonia* |
| 0.85555 | -0.01111 | *Blastomonas* |
| 0.85404 | -0.02711 | *Eubacterium* |
| 0.84703 | -0.01327 | *Neorickettsia* |
| 0.84682 | -0.00891 | *Rhodomicrobium* |
| 0.84081 | -0.00981 | *Methanoregula* |
| 0.83426 | -0.02471 | *Gordonibacter* |
| 0.82978 | -0.01305 | *Glarea* |
| 0.81529 | 0.02917 | *Rhinocladiella* |
| 0.80971 | -0.01014 | *Methylomicrobium* |
| 0.80387 | -0.01944 | *Pectobacterium* |

Table S6: Identity of MAGs carrying a specific gene and impacting on animal feed efficiency

| **KEGG ID** | **Genus MAG^1^** | **#MAG variant** | **IMPACT** |
| --- | --- | --- | --- |
| ko: K01654 | g__*Succinivibrio* | 11 | RFI+ |
| ko: K01840 | g__*Succiniclasticum* | 35 | FCR- |
| ko: K02005 | g__*Succiniclasticum* | 35 | FCR- |
| ko: K02283 | g__*Eubacterium*_A | 18 | RFI- |
| ko: K02283 | g__*Eubacterium*_B | 2 | RFI- |
| ko: K02283 | g__*Eubacterium*_E | 8 | RFI- |
| ko: K02283 | g__*Eubacterium*_F | 4 | RFI- |
| ko: K02283 | g__*Eubacterium*_G | 5 | RFI- |
| ko: K02283 | g__*Eubacterium*_H | 27 | RFI- |
| ko: K02283 | g__*Eubacterium*_I | 2 | RFI- |
| ko: K02283 | g__*Eubacterium*_Q | 38 | RFI- |
| ko: K02283 | g__*Eubacterium*_R | 1 | RFI- |
| ko: K02396 | g__*Eubacterium*_Q | 1 | RFI- |
| ko: K02410 | g__*Desulfovibrio* | 1 | RFI+ |
| ko: K02429 | g__*Lactobacillus*_H | 1 | FCR- |
| ko: K02454 | g__*Acidaminococcus* | 7 | RFI+/FCR+ |
| ko: K02652 | g__*Eubacterium*_A | 6 | RFI-/FCR+ |
| ko: K02652 | g__*Eubacterium*_E | 1 | RFI-/FCR+ |
| ko: K02652 | g__*Eubacterium*_F | 2 | RFI-/FCR+ |
| ko: K02652 | g__*Eubacterium*_H | 3 | RFI-/FCR+ |
| ko: K02652 | g__*Eubacterium*_Q | 40 | RFI-/FCR+ |
| ko: K02652 | g__*Eubacterium*_R | 1 | RFI-/FCR+ |
| ko: K02652 | g__*Treponema*_D | 13 | RFI-/FCR+ |
| ko: K02653 | g__*Acidaminococcus* | 6 | RFI+/FCR+ |
| ko: K03205 | g__*Treponema*_D | 6 | FCR+ |
| ko: K06442 | g__*Eubacterium*_A | 18 | RFI- |
| ko: K06442 | g__*Eubacterium*_B | 2 | RFI- |
| ko: K06442 | g__*Eubacterium*_E | 7 | RFI- |
| ko: K06442 | g__*Eubacterium*_F | 3 | RFI- |
| ko: K06442 | g__*Eubacterium*_G | 3 | RFI- |
| ko: K06442 | g__*Eubacterium*_H | 26 | RFI- |
| ko: K06442 | g__*Eubacterium*_I | 2 | RFI- |
| ko: K06442 | g__*Eubacterium*_Q | 30 | RFI- |
| ko: K06442 | g__*Eubacterium*_R | 13 | RFI- |
| ko: K11068 | g__*Bifidobacterium* | 9 | FCR- |

^1^Genera identified using the Genome Taxonomy Database (GitHub repository). GTDB.

+/-: Positive or negative correlation between a specific gene carried by an identified MAG with RFI, FCR or both.
