# Supplementary figures and images for: Identification of Microbial Genetic Capacities and Potential Mechanisms Within the Rumen Microbiome Explaining Differences in Beef Cattle Feed Efficiency
Source: Front Microbiol. 2020 Jun 5;11:1229. doi: 10.3389/fmicb.2020.01229 (PMC7292206; doi:10.3389/fmicb.2020.01229)

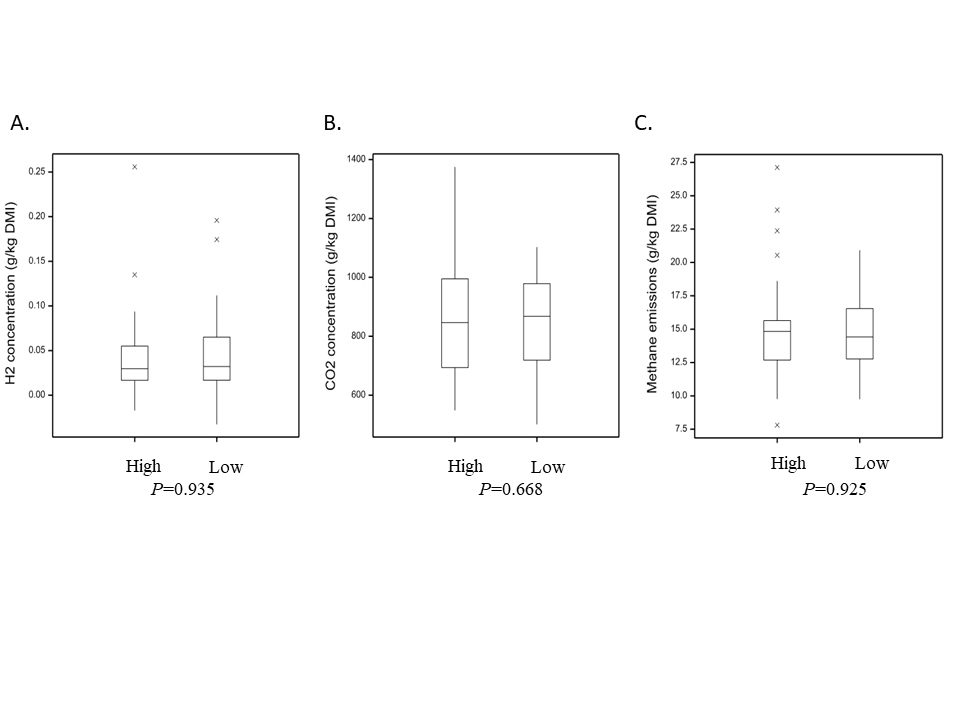

Supplement: Supplementary file 2 [file Data_Sheet_2.zip › Figure S1.TIF]

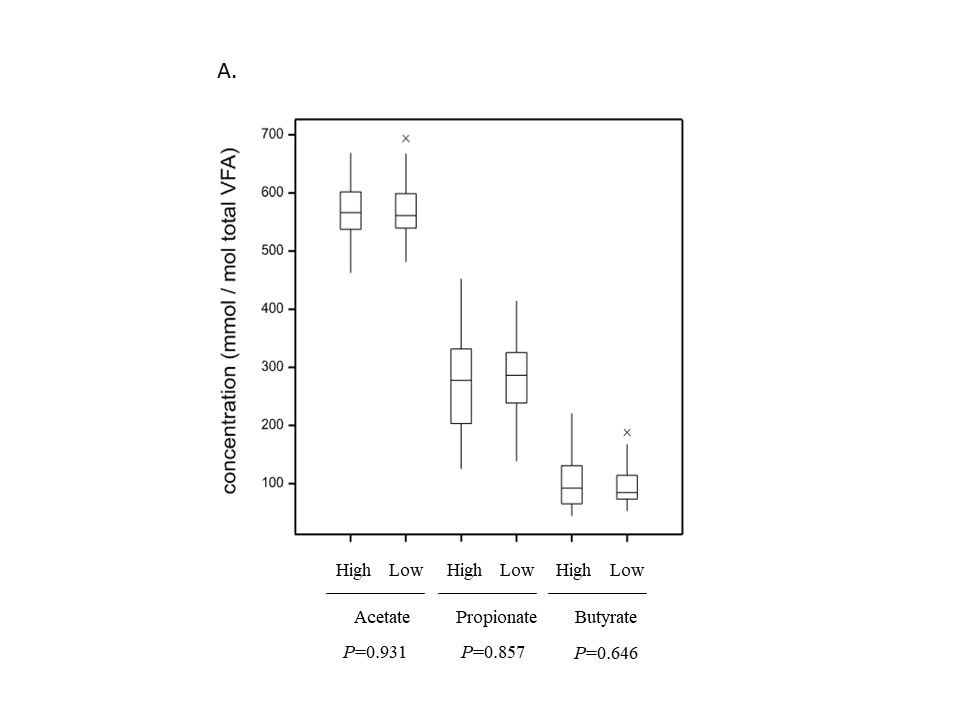

Supplement: Supplementary file 2 [file Data_Sheet_2.zip › Figure S2A.TIF]

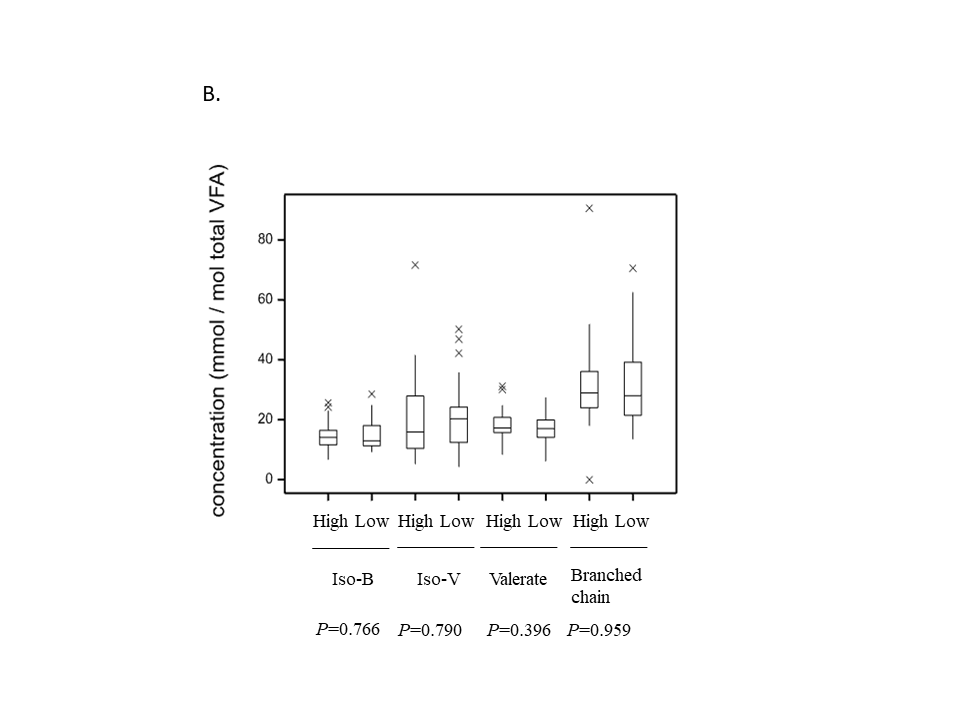

Supplement: Supplementary file 2 [file Data_Sheet_2.zip › Figure S2B.TIF]

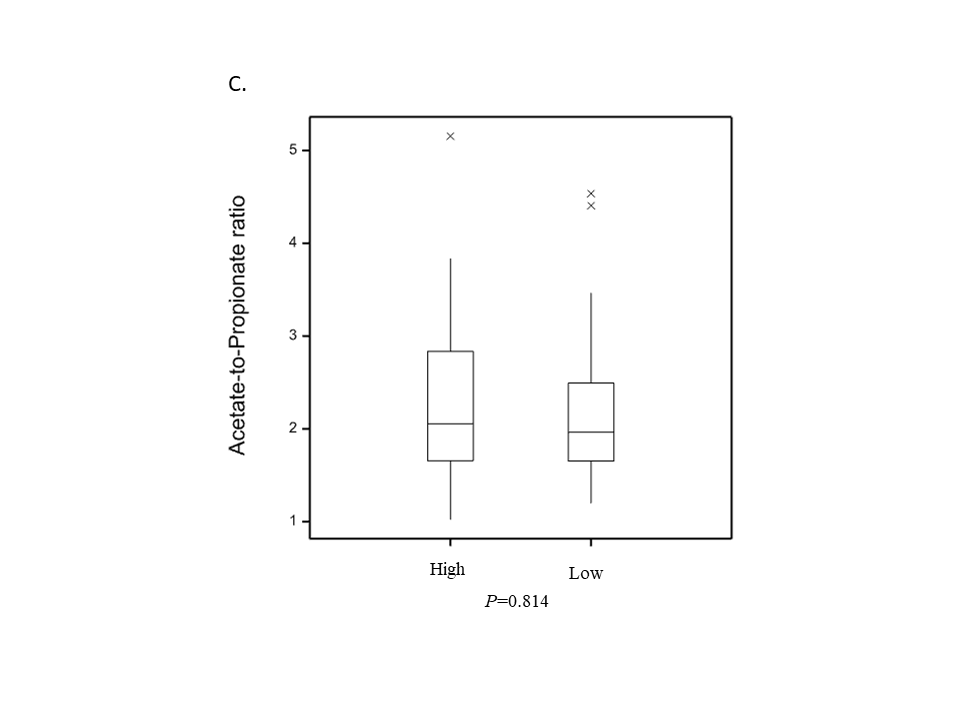

Supplement: Supplementary file 2 [file Data_Sheet_2.zip › Figure S2C.TIF]

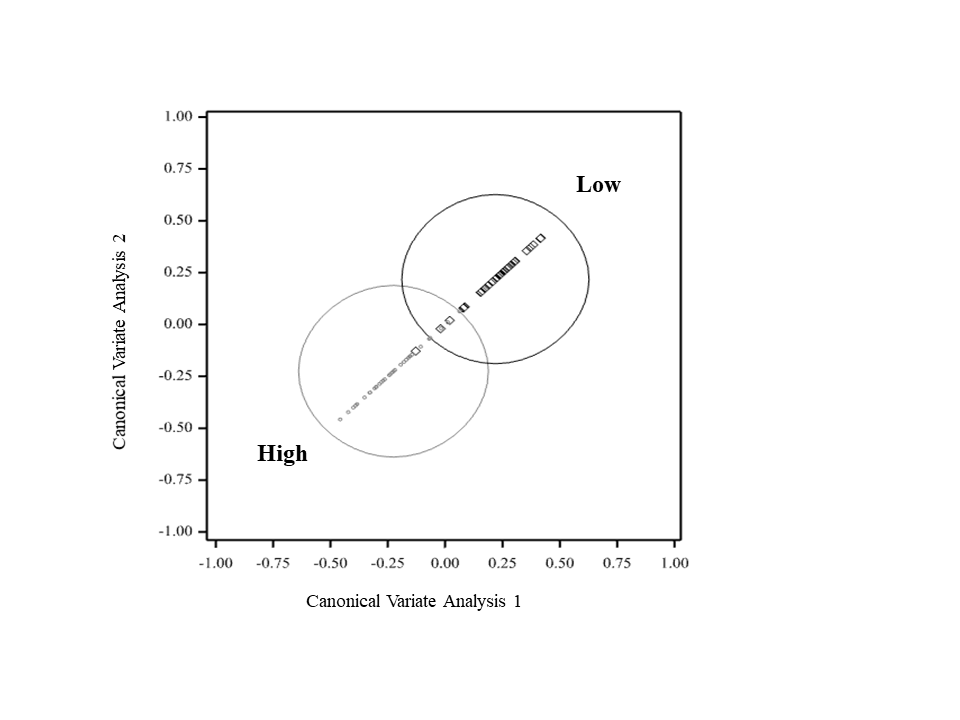

Supplement: Supplementary file 2 [file Data_Sheet_2.zip › Figure S3.TIF]

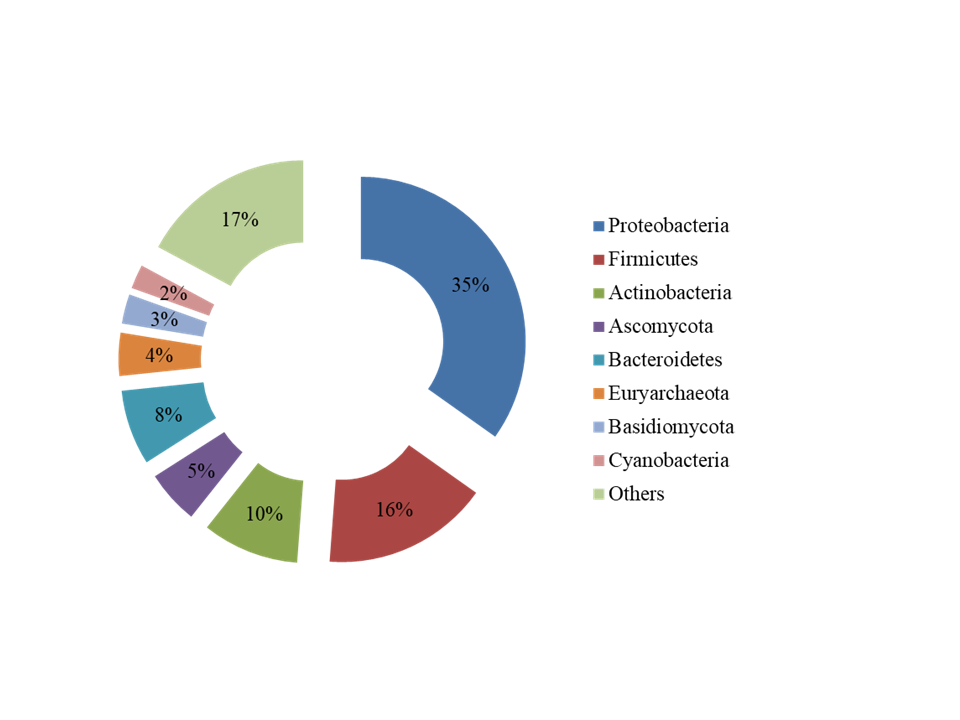

Supplement: Supplementary file 2 [file Data_Sheet_2.zip › Figure S4.TIF]
